# NEDD4L downregulates autophagy and cell growth by modulating ULK1 and a glutamine transporter

**DOI:** 10.1038/s41419-020-2242-5

**Published:** 2020-01-20

**Authors:** Dong-Eun Lee, Ju Eun Yoo, Jiyea Kim, Seungki Kim, Sunshin Kim, Ho Lee, Heesun Cheong

**Affiliations:** 1 Research Institute, National Cancer Center, 323 Ilsan-Ro, Ilsandong-Gu, Goyang-Si, Gyeonggi-Do Republic of Korea; 2 Department of Cancer Biomedical Science, Graduate School of Cancer Science & Policy, National Cancer Center, 323 Ilsan-Ro, Ilsandong-Gu, Goyang-Si, Gyeonggi-Do Republic of Korea

**Keywords:** Tumour-suppressor proteins, Macroautophagy

## Abstract

In mammals, autophagosome formation is initiated by ULK1 via the posttranslational modification of this protein. However, the precise role of ULK1 ubiquitination in modulating autophagy is unknown. Here, we show that NEDD4L, an E3 ubiquitin ligase, binds ULK1 in pancreatic cancer cells. ULK1 expression was stabilized in NEDD4L knockdown cells compared to that in control cells, suggesting that NEDD4L is involved in ULK1 ubiquitination and its subsequent degradation. Autophagy activity was enhanced in NEDD4L knockdown cells compared to control cells. NEDD4L-depleted cells exhibited an increase in the cellular oxygen consumption rate (OCR) and mitochondrial membrane potential, and maintained mitochondrial fusion status in response to metabolic stress. Enhanced OCR and mitochondrial fusion morphology in NEDD4L knockdown cells were repressed by siRNA targeting ULK1. In addition to ULK1, ASCT2, a glutamine transporter, was accumulated in NEDD4L-depleted cells; this is important for maintaining autophagy activation and mitochondrial metabolic function. Finally, the cellular growth and survival rate increased in NEDD4L knockdown cells compared to control cells. However, the genetic or pharmacological blockade of either ULK1 or ASCT2 in NEDD4L-depleted cells sensitized pancreatic cancer cells, particularly in response to nutrient deprivation. In a mouse xenograft model of pancreatic cancer, the use of autophagy inhibitors suppressed tumor growth more in NEDD4L-depleted cells than in tumors from control cells. NEDD4L and ULK1 levels were inversely correlated in two different pancreatic cancer mouse models-xenograft mouse and KPC mouse models. These results suggest that NEDD4L suppressed autophagy and mitochondrial metabolism by reducing cellular ULK1 or ASCT2 levels, and thus could repress the growth and survival of pancreatic cancer cells. Therefore, ubiquitin ligase-mediated autophagy plays a critical role in regulating mitochondrial metabolism, thereby contributing to the growth and survival of certain cancers with low NEDD4L levels.

## Introduction

Autophagy is a process that degrades unnecessary or damaged cellular components in lysosomes. Degradation products from autophagy can then be used as molecular precursors or cellular energy sources, which are important in times of cellular stress. Therefore, autophagy is induced by intra- and extracellular stressors as an adaptive survival mechanism^1,2^.

Autophagy has different features on the growth of cancer depending on the tumor type and stage of tumor development. Autophagy suppresses tumor growth in early tumor development through scavenging toxic molecules associated with tumorigenesis. In more advanced cancers, however, autophagy can promote tumor growth and survival to overcome cellular metabolic stress^3–6^.

Among the autophagy-related (ATG) genes, *ATG1* was the first identified ATG gene in yeast; its mammalian homolog, Unc51-like kinase 1 (ULK1), is a serine/threonine kinase that initiates autophagy in mammals. When the autophagy response is triggered, ULK1 forms a complex with three ATG proteins: ATG13, ATG101, and focal adhesion kinase (FAK) family interacting protein of 200 kDa (FIP200)^7,8^, through the phosphorylation of these interacting proteins, leading to the initiation of autophagy. The Vps34–Beclin1–ATG14 complex responsible for subsequent steps of autophagy is also regulated by ULK1 kinase activity through phosphorylation^8^. ULK1 activity is modulated by various posttranslational modifications^3,8,9^.

As a posttranslational modification, the ubiquitination of ULK1 is also important for regulating the autophagy pathway. ULK1 ubiquitination reduces the cellular levels of ULK1, thereby suppressing autophagy^10,11^. ULK1 ubiquitination is mediated by various autophagy proteins and E3 ubiquitin protein ligases, including the AMBRA1–TRAF6 complex, chaperone-like protein p32, and Cul3-KLHL20 ubiquitin ligase^11–13^. Multiple deubiquitinases (DUBs) are also involved in regulating ULK1 ubiquitination and stability^11–15^.

Neural precursor cell expressed developmentally downregulated 4-like (NEDD4L) is an E3 ubiquitin protein ligase that contains a HECT domain. Most identified targets of NEDD4L are membrane proteins, including ion channels and transporters. Given the crucial role of ion channels in maintaining homeostasis, the regulation of NEDD4L activity is important for maintaining blood pressure and normal physiology^16^. Some amino acid transporters have been identified as substrates of NEDD4L, although their physiological relevance is currently unclear^11–13,17^. NEDD4L also triggers the degradation of certain proteins involved in cancer signaling pathways, including disheveled-2 (Dvl2) and two mothers against decapentaplegic homolog (SMAD) proteins: SMAD2 and SMAD7. The degradation of Dvl2 results in the suppression of the Wnt signaling pathway^18,19^, while the degradation of SMAD2 and SMAD7 results in the down-regulation of transforming growth factor beta (TGF-β)^20,21^; both of which are closely related to the regulation of tumor progression.

Recently, Nazio et al.^22^ reported that NEDD4L directly regulates ULK1 ubiquitination and thereby modulates cellular autophagy. Despite the established role that NEDD4L plays in autophagy regulation through the regulation of ULK1 levels, it is not fully understood how NEDD4L directly alters cellular phenotypes through the modulation of ULK1 activity in terms of physiology.

Multiple cancer cell types express low levels of NEDD4L relative to normal cells^23–25^ indicating that NEDD4L potentially deregulates the stability of various proteins involved in tumor growth, thereby acting as a tumor suppressor^26^. However, in certain cancers, such as melanomas, tumor growth is inhibited when NEDD4L expression is suppressed^27^. Thus, the role of NEDD4L in cancer progression is complex and not yet fully understood.

Here, we investigate novel roles of NEDD4L in modulating autophagy activity and mitochondrial metabolism on contributing to tumor progression by which regulates the protein levels of an autophagy protein, ULK1, and ASCT2, a transporter of glutamine that is a substrate for mitochondrial anaplerosis.

## Results

### NEDD4L interacts with ULK1

**Fig. 1:**
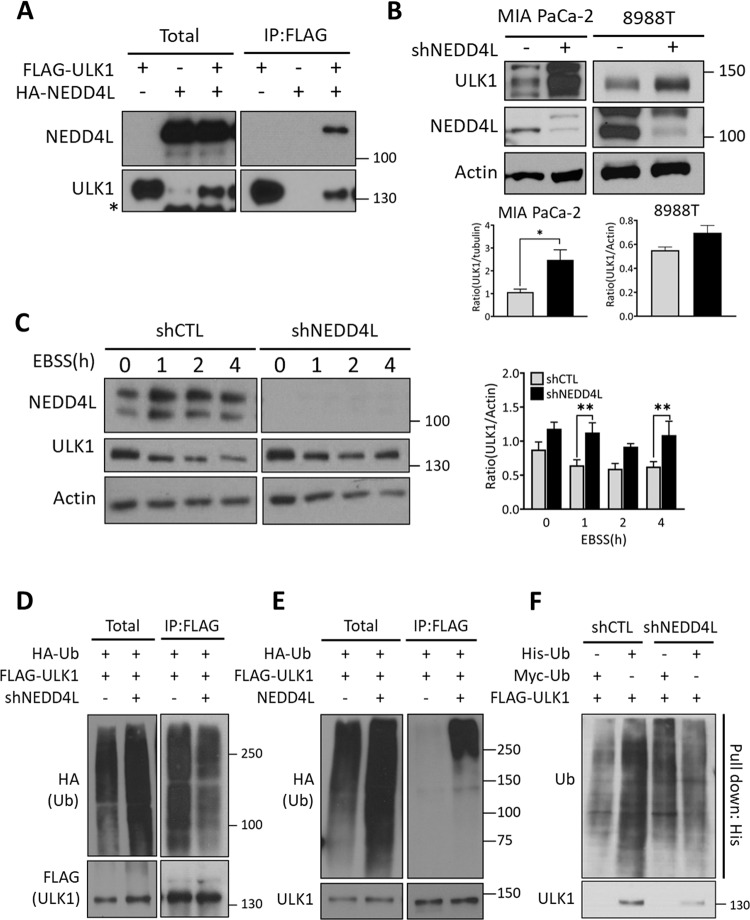
NEDD4L interacts with ULK1. **a** FLAG-ULK1 and/or Hemagglutinin (HA)-NEDD4L plasmid were expressed in HEK293T cells. Cells were treated with 10 μM MG132 for 1 h before lysis. Lysates were immunoprecipitated with anti-FLAG antibody and immunoblotted against NEDD4L and ULK1. **b** Cell lysates were prepared from MIA PaCa-2 and PA-TU 8988T stably expressing shCTL or shNEDD4L and immunoblotted against ULK1 and NEDD4L. β-actin was used as loading control. The WB band intensities were quantified by Image J and error bars indicate the mean ± SEM for three independent experiments. **c** HEK293T cells stably expressing shCTL or shNEDD4L were plated overnight and subsequently starved in EBSS media. Cells were collected and lysed at the indicated time points. Cell lysates were immunoblotted against NEDD4L and ULK1. ULK1 levels were normalized to β-actin which served as loading control. The WB band intensities were quantified by Image J and error bars indicate the mean ± SEM for three independent experiments. *P < 0.05; **P < 0.01. **d** HA-ubiquitin (HA-Ub) and FLAG-ULK1 plasmids were co-transfected into shCTL and shNEDD4L HEK293T cells, respectively. Cell lysates were immunoprecipitated with anti-FLAG antibody. ULK1 ubiquitination was determined by immunoblotting against HA and FLAG. **e** Immunoprecipitation of HA-ubiquitin (HA-Ub) and FLAG-ULK1 in HEK293T cells as in (**d**) was observed in WT cells and cells with NEDD4L ectopically overexpressed. **f** His-ubiquitin(His-Ub) or Myc-ubiquitin (Myc-Ub) was co-transfected with FLAG-ULK1 into shCTL or shNEDD4L MIA PaCa-2 cells, respectively. ULK1 ubiquitination was observed by pulldown of His-Ub using Ni-NTA beads and immunoblotting against Ubiquitin and ULK1.

NEDD4L, an E3 ubiquitin protein ligase, was identified as a candidate ULK1-interacting partner using immunoprecipitation combined with mass spectrometry (Table S1). Co-transfection of FLAG-tagged ULK1 with HA-tagged NEDD4L and subsequent immunoprecipitation revealed a band corresponding to the FLAG-ULK1 that co-immunoprecipitated with the HA-NEDD4L (Fig. 1a), indicating that NEDD4L binds to ULK1.

### NEDD4L deregulates the stability of ULK1 via ubiquitination activity

As NEDD4L, a newly identified ULK1-interacting protein, is a well-known E3 ubiquitin protein ligase, the modulation of ULK1 levels in the presence or absence of NEDD4L were next examined in pancreatic cancer cell lines.

NEDD4L knockdown cell lines (shNEDD4L) were generated using a lentiviral vector containing small hairpin RNA (shRNA) targeting NEDD4L, ULK1 protein levels were compared by immunoblot analysis between shNEDD4L cells and shControl (shCTL) cells, which were infected with a vector containing scramble shRNA, and revealed that ULK1 protein levels were higher in shNEDD4L cells compared to shCTL cells (Figs. 1b and S1). Furthermore, we examined whether NEDD4L would regulate ULK1 stability by measuring the protein levels of ULK1 in shNEDD4L and shCTL in a nutrient-deprived conditions. ULK1 protein levels were substantially decreased in shCTL cells, but remained relatively stable in shNEDD4L cells (Fig. 1c), indicating that NEDD4L disrupts ULK1 levels during autophagy-inducing conditions. However, NEDD4L depletion did not affect the expression levels of ATG13 and Beclin1 which are essential components for autophagy initiation (Fig. S2).

Next, we investigated whether ULK1 stability would be affected by the ubiquitin ligase activity of NEDD4L. shCTL cells showed relatively higher ubiquitin levels than shNEDD4L cells on the immunoblot analysis of the immunoprecipitated complex with anti-FLAG (Fig. 1d). Moreover, immunoblot analysis of the ULK1-immunoprecipitated complex indicated that the ectopic expression of NEDD4L increased ubiquitination levels of ULK1 compared to the empty vector-expressed condition (Fig. 1e). In addition, we performed Ni-NTA pull-down assays after expressing His-Ubiquitin (His-Ub) in shNEDD4L and shCTL cells, indicating that ULK1 levels were markedly decreased in the shNEDD4L cell fractions compared to those in shCTL fractions (Fig. 1f). The present results suggest that NEDD4L plays a negative role in maintaining ULK1 protein stability through the ubiquitination of ULK1.

### NEDD4L depletion stimulates autophagy activity

**Fig. 2:**
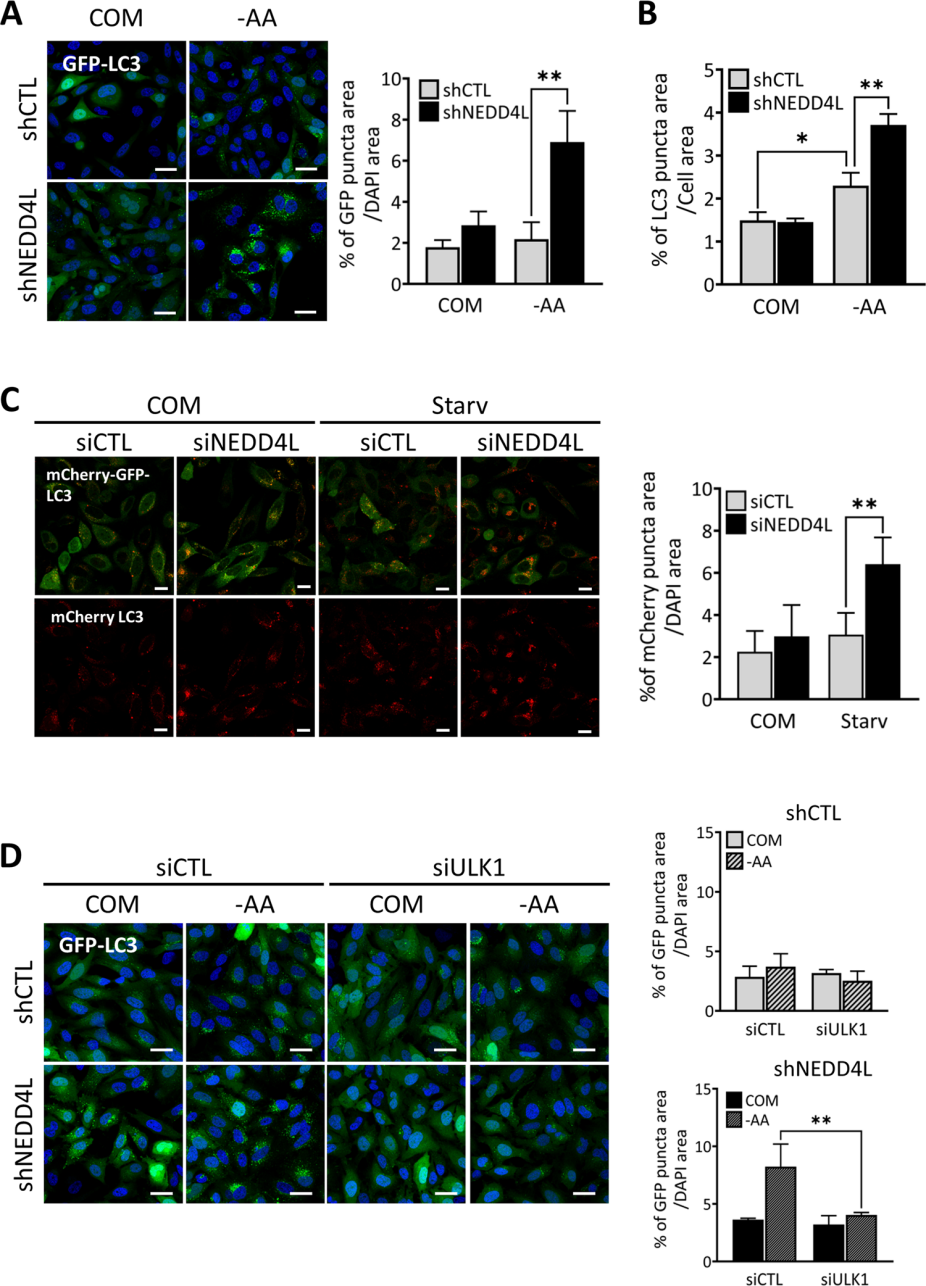
NEDD4L depletion increases autophagy activity. **a** shCTL or shNEDD4L MIA PaCa-2 cells stably expressing GFP-LC3 were plated overnight and subsequently starved in amino acids-deprived media(-AA) for 4 h. Cell nuclei were stained with Hoechst 33342 or DAPI, and images were acquired using a confocal fluorescence microscope. At least five distinct regions were imaged per condition and quantified. Scale bar: 20 μm (Magnification, ×400). Percent of GFP-LC3 puncta area were normalized to the DAPI-stained area per cell. Error bars indicate the mean ± SEM for three independent experiments. *P < 0.05; **P < 0.01. **b** GFP-LC3 puncta in shCTL or shNEDD4L HeLa cells stably expressing GFP-LC3 was imaged up to 8 h in amino acids-deprived media and quantified with a high-content screening system. Error bars indicate the mean ± SEM for three independent experiments. *P < 0.05; **P < 0.01. **c** Hela cells stably expressing mCherry-GFP-LC3 were reverse-transfected with either scrambled siRNA (siCTL) or NEDD4L-specific siRNA (siNEDD4L), respectively for 24–48 h. Subsequently, cells were incubated in nutrient-complete or amino acids-deprived medium(Starv) for 2 h prior to image acquisition using a confocal fluorescence microscope. Scale bar: 20 μm (Magnification, ×400). Percent of mCherry puncta area was quantified and normalized to the DAPI-stained area per cell. The data are representative of at least three independent experiments. Error bars indicate the mean ± SEM for three independent experiments. *P < 0.05; **P < 0.01. **d** shCTL and shNEDD4L HeLa cells stably expressing GFP-LC3 plasmid were reverse-transfected with either scramble siRNA (siCTL) or ULK1-specific siRNA (siULK1). Subsequently, cells were incubated in nutrient-complete or amino acids-deprived medium for 4 h prior to image acquisition. At least five distinct regions were imaged per sample. Scale bar: 20 μm (Magnification, ×400). Percent of GFP-LC3 puncta area was normalized to the DAPI-stained area per cell. The data are representative of at least three independent experiments. Error bars indicate the mean ± SEM for three independent experiments. *P < 0.05; **P < 0.01.

Because ULK1 protein is important for autophagy, we next examined whether NEDD4L would influence autophagy activity. Immunoblot analysis of shNEDD4L cells revealed higher levels of LC3-II in both nutrient-rich and -deprived conditions compared to shCTL cells. Moreover, when cells were cultured in nutrient-deprived conditions (Earle’s Balanced Salt Solution (EBSS)), shNEDD4L cells exhibited further increases in LC3-II levels compared to those in shCTL cells, suggesting that NEDD4L suppresses autophagy (Fig. S3). Next, we examined autophagy activity by monitoring changes in the subcellular localization of green fluorescent protein (GFP)-tagged LC3 through confocal fluorescence microscopy. Under autophagy-inducing conditions, such as nutrient starvation, cytoplasmic GFP-LC3 conjugates to the autophagosome membrane, and exhibits a distinct punctate form, indicating the activation of autophagy. A substantial increase in GFP-LC3 puncta formation was seen in shNEDD4L cells compared to shCTL cells under both nutrient-rich and -deprived conditions. Quantification of GFP-LC3 puncta per cell by normalizing the percent of green puncta areas (GFP-LC3) by the total area of DAPI-stained cells which indicated that GFP-LC3 puncta levels were higher in shNEDD4L cells than in shCTL cells (Fig. 2a). A GFP-LC3 puncta assay was similarly performed using an image-based high contents screening (HCS) system. The area of GFP-LC3 puncta relative to the total area of cells was substantially increased in response to nutrient deprivation, and was further increased in NEDD4L-knockdown cells (Fig. 2b). Moreover, tandem mCherry-GFP-LC3 was observed through confocal microscopy to represent autophagy flux. mCherry signals through lysosome-mediated autophagic degradation were significantly increased in siNEDD4L cells compared to that in siCTL cells, indicating autophagy flux was enhanced by NEDD4L depletion (Fig. 2c). The data comprehensively suggest that NEDD4L acts as a negative regulator of autophagosome formation.

Finally, when ULK1 was suppressed by siRNA in both shCTL and shNEDD4L cells, the increase in GFP-LC3 puncta formation in shNEDD4L cells regressed to levels similar to those in shCTL cells under nutrient-deprived conditions (Fig. 2d), suggesting that NEDD4L depletion enhanced autophagy activity mediated by ULK1.

### NEDD4L depletion increases mitochondrial respiratory functions

**Fig. 3:**
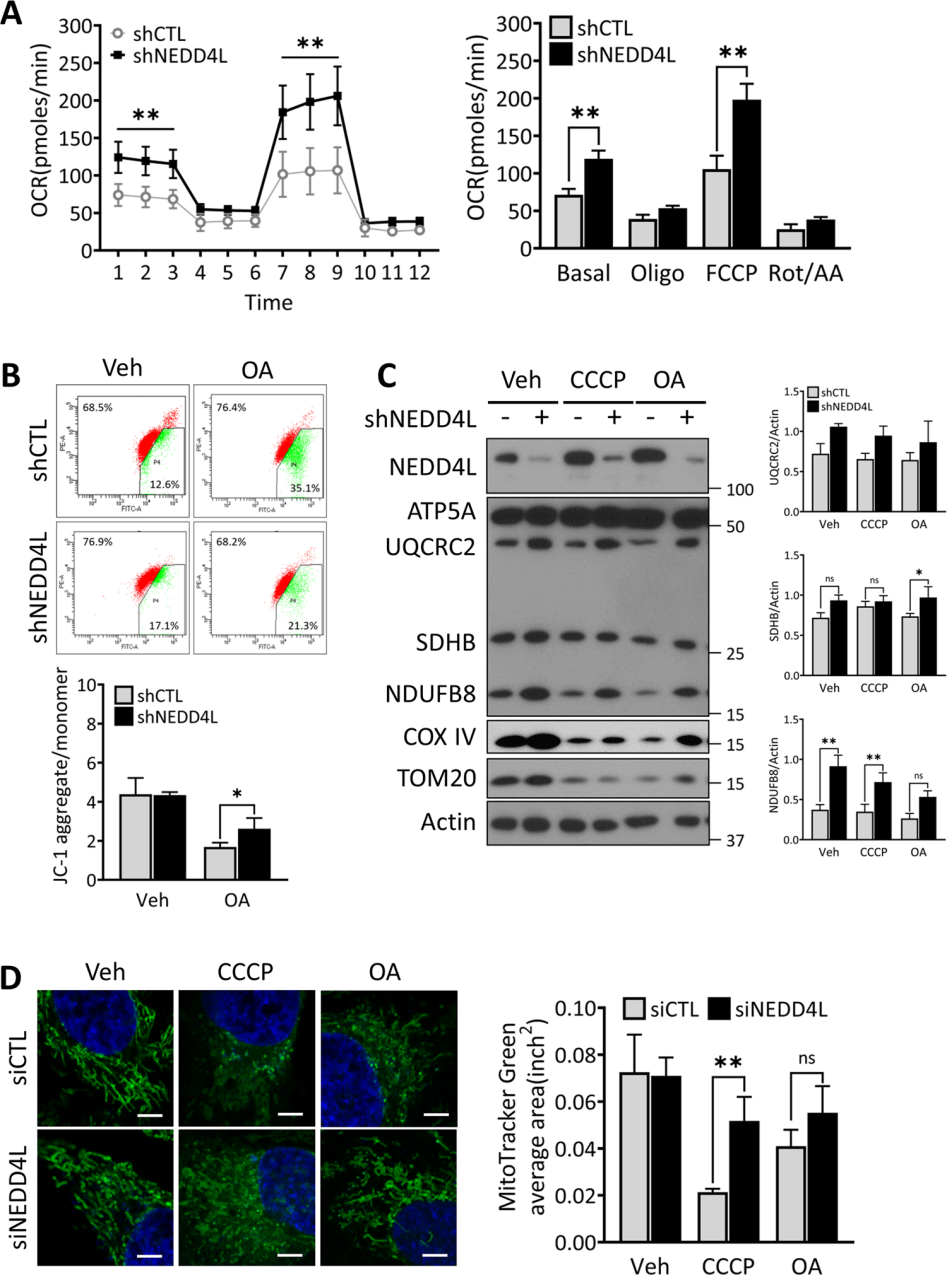
Mitochondrial respiration increases in NEDD4L-deficient cells. **a** Mitochondrial oxygen consumption rate (OCR) of MIA PaCa-2 shCTL and shNEDD4L cells was measured by an extracellular flux analyzer in basal media and in response to treatment with the indicated regulators of mitochondrial activities, including 1 μM Oligomycin, 0.5 μM FCCP, and 0.45 μM Rotenone/Antimycin A, for the indicated time periods. The levels of OCR were normalized by cell numbers which were measured by image-based HCS system Operetta CLS immediately after the analysis. *P < 0.05; **P < 0.01. **b** Cells as in (**a**) were plated overnight and subsequently treated with vehicle (DMSO) or Oligomycin/Antimycin A (OA; 1 μM/3 μM) for 2 h to induce mitochondrial dysfunction. Subsequently, cells were stained with JC-1 mitochondrial dye and analyzed by flow cytometry. Error bars indicate the mean ± SEM for three independent experiments. *P < 0.05; **P < 0.01. **c** Cells as in (**a**) were plated overnight and subsequently treated with vehicle (DMSO), CCCP (10 μM), or Oligomycin/Antimycin A (OA; 1 μM/3 μM), respectively for 4 h and resolved in a SDS-PAGE gel. Samples were immunoblotted against NEDD4L, COXIV, TOM20, and a cocktail antibody of oxidative phosphorylation (OXPHOS) complex members. β-actin was used as loading control. The WB band intensities were quantified by Image J and error bars indicate the mean ± SEM for three independent experiments. *P < 0.05; **P < 0.01. **d** HeLa cells were reverse-transfected with either siCTL or siNEDD4L and were treated with vehicle (DMSO), CCCP (10 μM), or OA (1 μM/3 μM) for 1 h. Subsequently, the cells were stained with MitoTracker Green to visualize subcellular mitochondrial morphology using a confocal fluorescence microscope. Scale bar: 5 μm (Magnification, ×1000). The average area of MitoTracker Green signal was quantified and presented as an average from at least five different images in three independent experiments. **P < 0.01.

Since ULK1-mediated autophagy has been reported to be critical for the maintenance of mitochondrial homeostasis^28^, we next examined whether NEDD4L depletion would affect the function of mitochondrial respiration. shNEDD4L cells were monitored in an extracellular flux analyzer, an equipment measuring mitochondrial oxygen consumption rate (OCR), to study the extent of mitochondrial oxidative phosphorylation (OXPHOS) in cultured cells. The OCR in shNEDD4L cells was significantly higher than that in shCTL cells (Fig. 3a). Both basal and maximal OCR (after carbonyl cyanide-4-(trifluoromethoxy) phenylhydrazone [FCCP] treatment) in shNEDD4L cells were higher than that in shCTL cells, suggesting that mitochondrial OXPHOS in shNEDD4L cells was substantially enhanced compared to that in shCTL cells (Fig. 3a). In addition, JC-1 reagent was used to assess the mitochondrial membrane potential (MMP) in shNEDD4L and shCTL, showing a ratio of JC-1 aggregate/monomer. Results demonstrate that shNEDD4L cells showed relatively higher MMP than shCTL cells, especially under chemical inhibition of the mitochondrial electron transport chain. The ability of shNEDD4L cells to maintain MMP was consistently observed in various pancreatic cancer cells (Figs. 3b and S4).

Considering the maintenance of MMP is mediated by mitochondrial OXPHOS complexes, we measured the protein levels of mitochondrial OXPHOS complexes in shNEDD4L and shCTL cells. Protein levels of mitochondrial OXPHOS complex I (NDUF8B), complex II (succinate dehydrogenase complex iron sulfur subunit B [SDHB]), complex III (UQCRC2) members in shNEDD4L cells were found to be higher than those in shCTL cells. The accumulation of OXPHOS complex proteins in shNEDD4L cells was substantially higher compared to that in shCTL cells even in treatment with OXPHOS inhibitors such as carbonyl cyanide m-chlorophenyl hydrazone (CCCP) or Oligomycin/Antimycin A(OA) (Figs. 3c and S5).

Moreover, we examined whether NEDD4L would influence the morphology of mitochondria. Subcellular mitochondrial morphology in shNEDD4L and shCTL cells was observed using an airy-scan confocal microscope and revealed that NEDD4L knockdown cells contained longer mitochondria with more intact fusion states compared to shCTL cells, especially in response to CCCP (Fig. 3d). These results suggest that the MMP and intact morphology of mitochondria were more consistently maintained in NEDD4L knockdown cells than in control cells.

### NEDD4L depletion stabilizes glutamine transporters

**Fig. 4:**
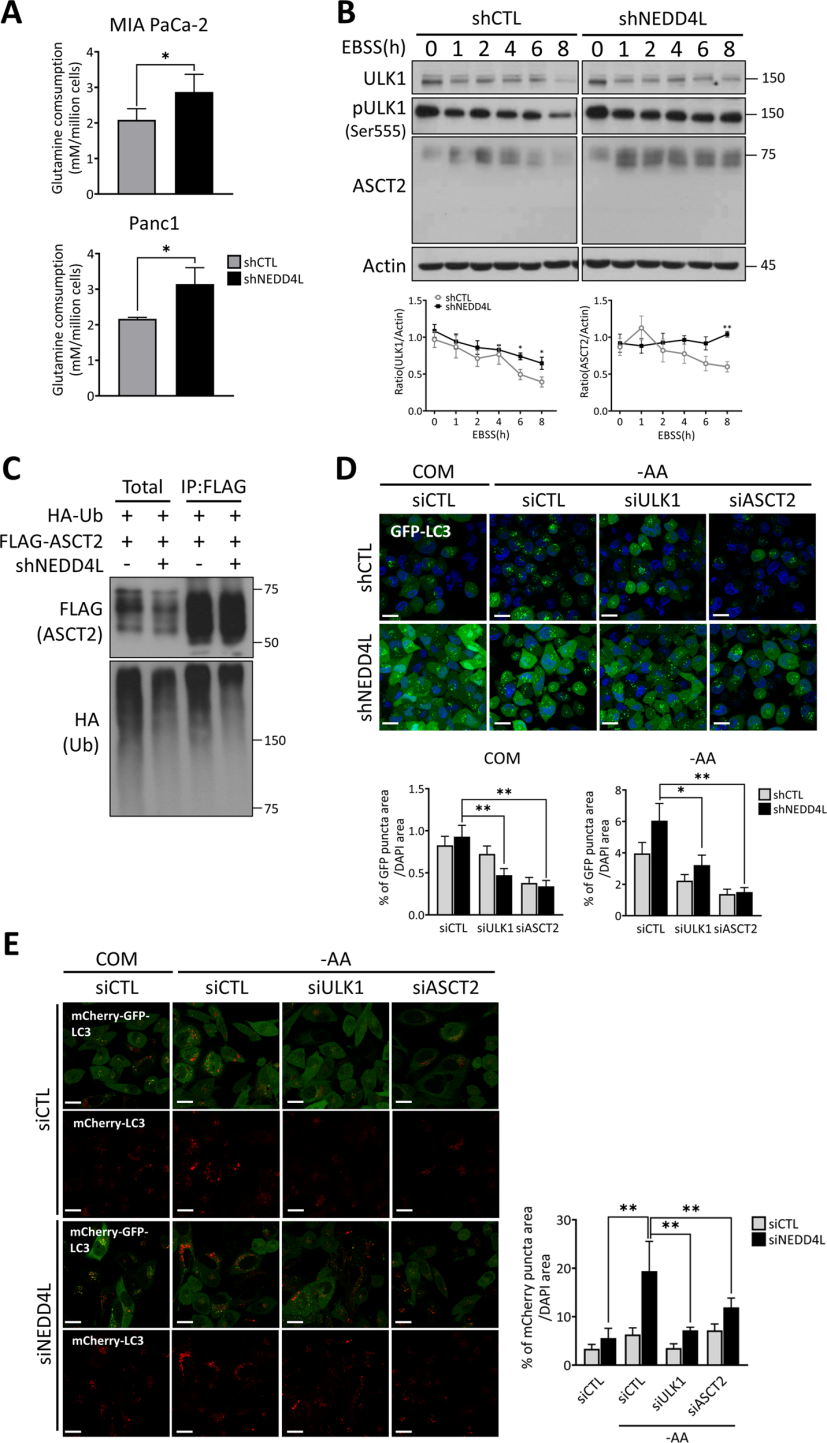
NEDD4L depletion stabilizes the glutamine transporter ASCT2. **a** MIA PaCa-2 and Panc-1 cells stably expressing shCTL and shNEDD4L were cultured for 24 h after which the culture media was collected and cells enumerated. Glutamine consumption levels of the cells were analyzed by comparing glutamine levels from media alone without cells and the collected cell culture media using a metabolite analyzer. The glutamine consumption rate was normalized to the number of cells. Error bars indicate the mean ± SEM for three independent experiments. *P < 0.05. **b** shCTL and shNEDD4L MIA PaCa-2 cells were plated overnight and subsequently replaced with EBSS media for starvation. Cell lysates were prepared at the indicated time points and immunoblotted with antibodies against ULK1, pULK1, and ASCT2. β-actin was used as loading control. The WB band intensities were quantified by Image J and error bars indicate the mean ± SEM for three independent experiments. *P < 0.05; **P < 0.01. **c** HA-ubiquitin and FLAG-ASCT2 plasmids were co-transfected in HEK293T shCTL and shNEDD4L cells for 48 h before harvest. Cell lysates were immunoprecipitated with anti-FLAG antibody and immunoblotted against FLAG and HA to observe ASCT2 ubiquitination. **d** shCTL and shNEDD4L MIA PaCa-2 cells stably expressing GFP-LC3 were reverse-transfected with control siRNA (siCTL), ULK1-specific siRNA (siULK1), or ASCT2-specific siRNA (siASCT2) for 48 h. Subsequently, cells were incubated in either nutrient-complete or amino acids-deprived medium for 4 h and imaged using a confocal fluorescence microscope. Scale bar: 20 μm (Magnification, ×400). Percent of GFP-LC3 puncta area was normalized to the DAPI-stained area per cell. The data are representative of at least three independent experiments. Error bars indicate the mean ± SEM for three independent experiments. *P < 0.05; **P < 0.01. **e** HeLa cells stably expressing mCherry-GFP-LC3 were reverse-transfected with siCTL, siULK1, or siASCT2 in addition to siCTL and siNEDD4L respectively. Cells were imaged under starvation as in (**d**). Percent of mCherry puncta area was normalized to the DAPI-stained area per cell. The data are representative of at least three independent experiments. Error bars indicate the mean ± SEM for three independent experiments. *P < 0.05; **P < 0.01.

To further investigate the mechanism by which NEDD4L deregulates mitochondrial function, we then investigated whether glutamine metabolism would be deregulated by NEDD4L to influence mitochondrial respiration. It has been previously reported that glutamine is essential for cell survival in pancreatic cancer through its utilization as a critical amino acid for either the maintenance of redox balance or anaplerosis replenishment during the tricarboxylic acid cycle in mitochondria^18,29^. Therefore, we predicted that proteins with a role in glutamine transport or metabolism would also be regulated by NEDD4L. First, glutamine uptake was investigated in the absence of NEDD4L in pancreatic cancer cells using an extracellular metabolite analyzer. Compared to shCTL cells in the same culture conditions, shNEDD4L cells showed an increase in the uptake of extracellular glutamine, despite of the constant levels of glucose uptake and lactate secretion. This data implies that NEDD4L might be involved in the downregulation of extracellular glutamine uptake from the media (Figs. 4a and S6). Next, we examined the protein levels of a glutamine transporter, ASCT2 in the presence or absence of NEDD4L. The levels of ASCT2 were markedly decreased in shCTL cells upon nutrient starvation whereas they were significantly stabilized in shNEDD4L cells (Figs. 4b and S7). To determine whether ASCT2 ubiquitination is affected by NEDD4L, FLAG-ASCT2, and HA-Ubiquitin plasmids were co-expressed in shNEDD4L cells and shCTL cells respectively, and immunoprecipitated with anti-FLAG antibody. Immunoblot analysis revealed relatively lower ubiquitin levels of ASCT2 in shNEDD4L cells compared to shCTL cells (Fig. 4c).

Finally, we examined whether ASCT2 played a role in in autophagy activated by NEDD4L knockdown. Interestingly, the increase in both GFP-LC3 puncta and mCherry puncta signal from mCherry-GFP-LC3 observed in NEDD4L depletion was markedly reduced when siNEDD4L cells were treated with siASCT2, as seen with confocal microscopy. This phenotype was similar to that observed in ULK1 knockdown cells (Fig. 4d, e). These results suggest that ASCT2 plays a crucial role in activating autophagy, particularly under NEDD4L-depleted conditions.

### NEDD4L downregulation stimulates mitochondrial OXPHOS via ULK1 or glutamine transporter

**Fig. 5:**
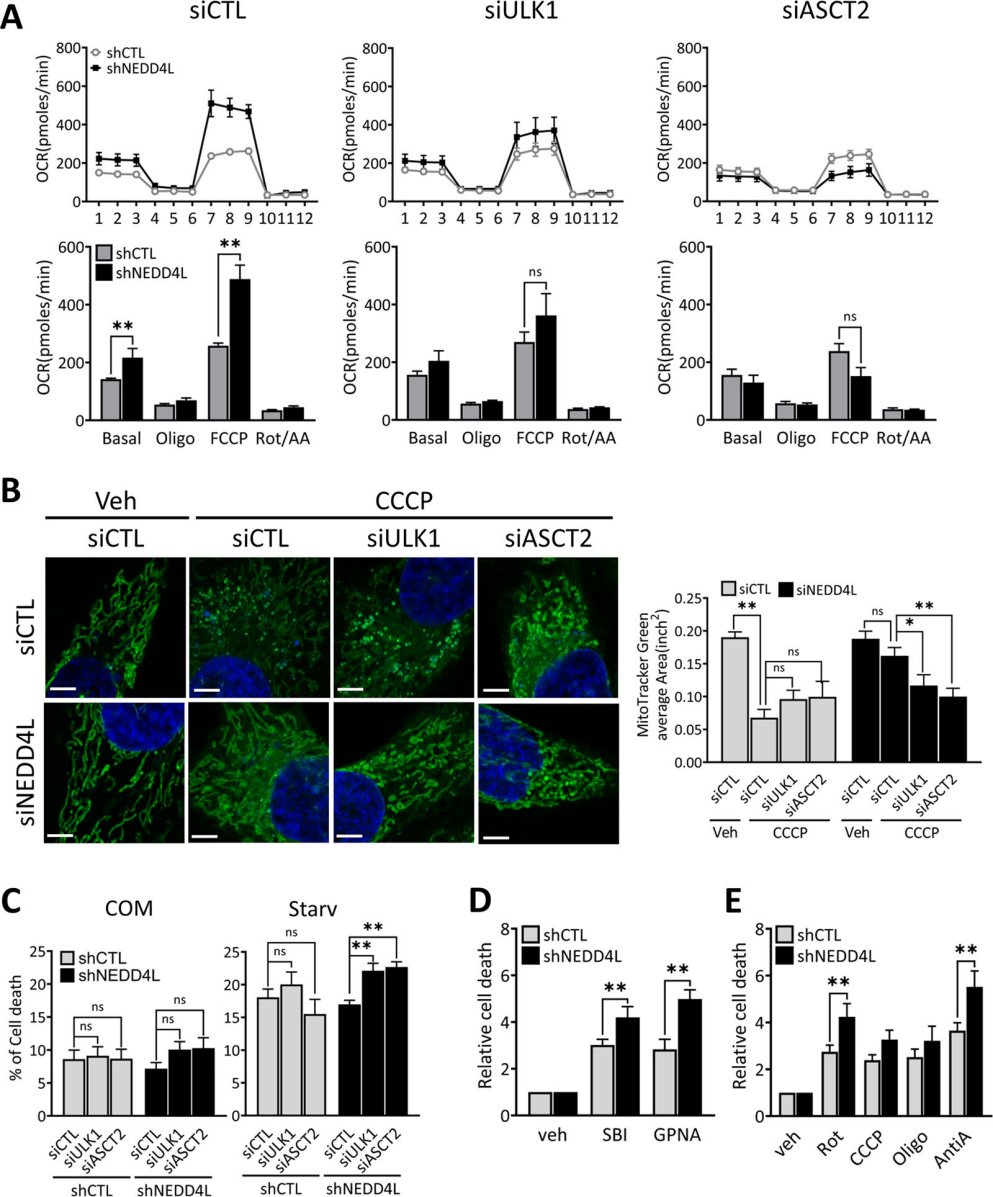
NEDD4L depletion stimulates mitochondrial oxidative phosphorylation and cell survival via regulation of ULK1 or glutamine transporter ASCT2. **a** shCTL and shNEDD4L MIA PaCa-2 cells were reverse-transfected with siCTL, siULK1, or siASCT2 and incubated for 48 h. The levels of mitochondrial OCR in each knockdown were measured by an extracellular flux analyzer in basal media and in response to treatment with the indicated mitochondrial regulators. *P < 0.05; **P < 0.01. **b** HeLa cells were reverse-transfected with siCTL, siULK1, or siASCT2 in siCTL and siNEDD4L respectively. Cells were stained with MitoTracker Green and then treated with either vehicle (DMSO) or CCCP (5 μM) for 1 h. Subcellular mitochondrial morphology were imaged using a confocal fluorescence microscope. Scale bar: 5 μm (Magnification, ×1000). The average area of MitoTracker Green was quantified. The data are representative of at least three independent experiments. Error bars indicate the mean ± SEM for three independent experiments. *P < 0.05; **P < 0.01. **c** shCTL and shNEDD4L MIA PaCa-2 cells were reverse-transfected with siCTL, siULK1, or siASCT2 for 24 h. Cells were then incubated in nutrient-complete media or glutamine-free (Starv) media for 48 h. Cell death was assessed using annexin V/propidium iodide (PI) staining by flow cytometry. The dead cell portion was measured based on the number of annexin V and PI single-stained cells. Error bars indicate the mean ± SEM for three independent experiments. **P < 0.01. **d** shCTL and shNEDD4L MIA PaCa-2 cells were treated with vehicle (0.5% DMSO), SBI 0206965 (ULK1 inhibitor), or GPNA (ASCT2 inhibitor) for 48 h. Cell death rate was analyzed as in (**c**) and normalized against death from vehicle, respectively. Error bars indicate the mean ± SEM for three independent experiments. **P < 0.01. **e** Cells as in (**d**) were treated with vehicle (0.5% DMSO), and the indicated mitochondrial OXPHOS inhibitors: rotenone, CCCP, oligomycin, and antimycin A. Cell death was analyzed as in (**c**). Error bars indicate the mean ± SEM for three independent experiments. **P < 0.01.

When shNEDD4L cells were treated with siULK1 or siASCT2, the increased mitochondrial OCR observed in untreated NEDD4L-depleted cells was significantly reduced to levels similar to those in shCTL cells. In particular, the maximum OCR in shNEDD4L cells treated with FCCP was more notably diminished by the knockdown of either ULK1 or ASCT2 (Fig. 5a). Higher MMP observed in shNEDD4L upon inhibition of mitochondrial OXPHOS, represented by a higher ratio of JC-1 aggregate/monomer (Fig. 3b), which was significantly decreased when there was an additional knockdown of either ULK1 or ASCT2 (Fig. S8). These results indicate that the dysregulation of mitochondrial OXPHOS by NEDD4L ultimately requires the expression of autophagy machinery including ULK1 or the glutamine transporter ASCT2.

Furthermore, mitochondrial morphology that was sustained by the knockdown of NEDD4L was significantly affected by the additional knockdown of either ULK1 or ASCT2 (Fig. 5b). These results indicate that the increased mitochondrial functional status in NEDD4L-depleted cells might be caused by the restoration of intact mitochondria through the autophagic degradation. ULK1 or ASCT2, stabilized by the loss of NEDD4L, play a critical role in autophagy activity, in addition to the direct function of ASCT2 in transporting key substrates for mitochondrial metabolism.

### NEDD4L negatively regulates the growth and survival of pancreatic cancer cells

Based on the above results, we hypothesized that NEDD4L should play a role as a tumor suppressor in pancreatic cancer cells through the deregulation of autophagy and mitochondrial function. To investigate this hypothesis, we examined whether NEDD4L depletion would influence cell growth or survival in pancreatic cancer cells. An image-based cell proliferation analyzer was used to assess the proliferation of shNEDD4L cells and shCTL cells. It was found that the growth rate of NEDD4L-knockdown conditions was higher than control in various pancreatic cancer cells. Moreover, the growth rate of shNEDD4L cells was significantly suppressed by the ectopic expression of NEDD4L (Fig. S9), suggesting that NEDD4L negatively regulates cancer cell proliferation.

When shNEDD4L cells were additionally absent of either ULK1 or ASCT2 expression by siRNA treatment, cell death in shNEDD4L cells under nutrient deprivation was markedly increased compared to that in shCTL cells (Fig. 5c). Similarly, treatment of cells with pharmacological inhibitors SBI-0206965 (ULK1 inhibitor) or GPNA (ASCT2 inhibitor) caused a significant increase in shNEDD4L cell death rate (Fig. 5d). Moreover, treatment with pharmacological inhibitors for mitochondrial OXPHOS complex components such as Rotenone (complex I inhibitor), CCCP (mitochondrial uncoupler), Oligomycin (complex V inhibitor) and Antimycin A (complex III inhibitor) also substantially elevated cell death rate in shNEDD4L cells compared to that in shCTL cells (Fig. 5e). These data indicate that the depletion of NEDD4L enhanced mitochondrial integrity through the elevation of ULK1 or ASCT2 protein activity, thereby preventing cell death due to metabolic stress.

### NEDD4L suppresses pancreatic tumor growth in a mouse xenograft model

**Fig. 6:**
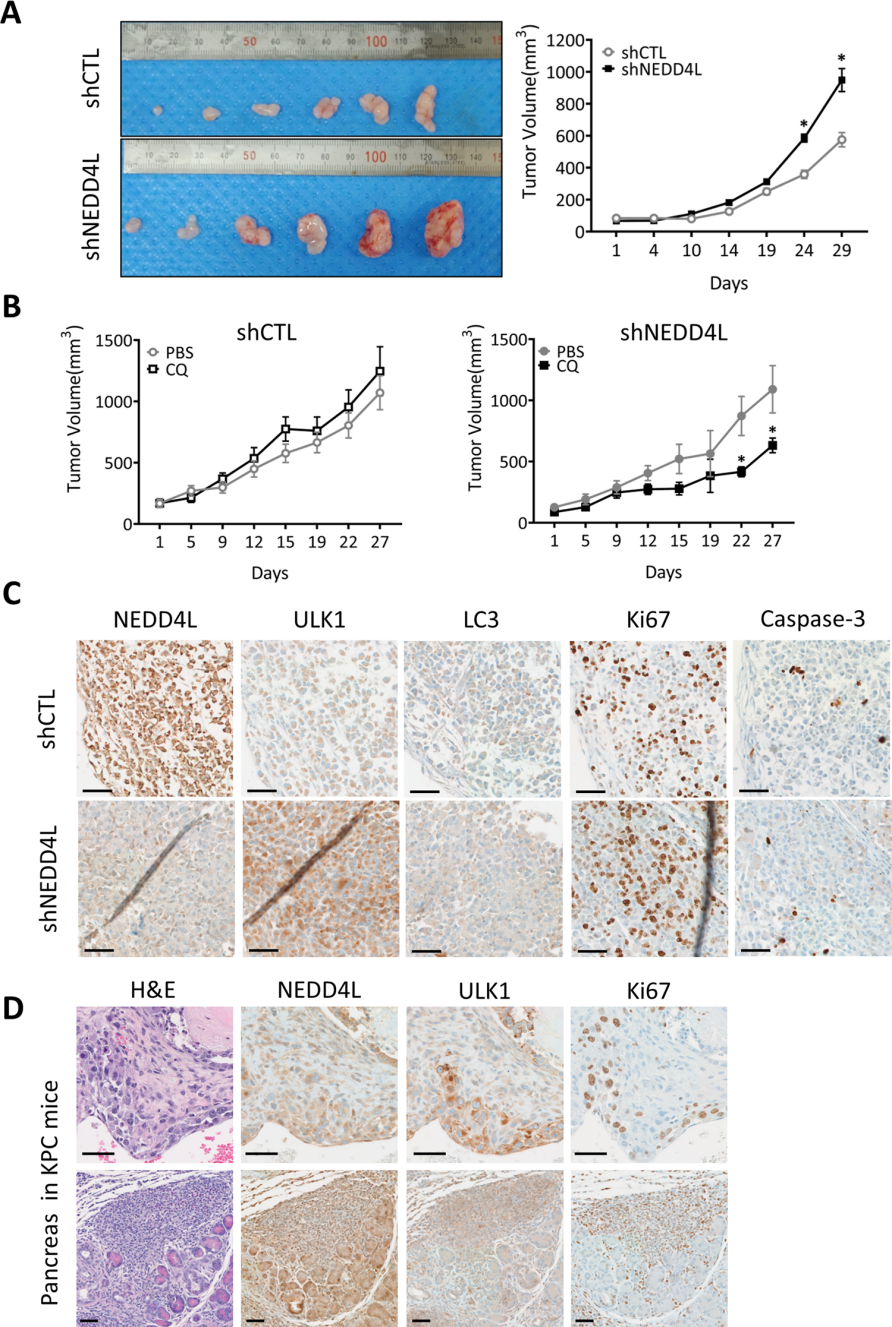
NEDD4L represses pancreatic cancer growth in xenograft mouse models by inactivation of autophagy. **a** Nude mice were injected subcutaneously with 5 × 10^6^ shCTL or shNEDD4L MIA PaCa-2 cells. When the average tumor size reached 40 mm^3^, tumor size was measured at the indicated time points. After 4 weeks, the tumors were removed for morphological and histochemical examinations. Representative images of tumors from shCTL (top) and shNEDD4L cells (bottom) are shown. *P < 0.05. **b** Subcutaneous shCTL or shNEDD4L MIA PaCa-2 cell-derived tumors were established in 6-week old female mice. Then, chloroquine (CQ; 20 mg kg^−1^) was administered daily via intraperitoneal injection and tumor growth was assessed once the tumor volume reached 100 mm^3^ at the indicated time points. Data are shown as the mean of five mice in each group ± SEM. *P < 0.05. **c** Immunohistochemistry of NEDD4L and ULK1 proteins detected from tumor tissues in (**a**). Tumors derived from shCTL or shNEDD4L MIA PaCa-2 cells were dissected 6 weeks after cell inoculation and embedded in paraffin, and tumor sections were stained with antibodies against NEDD4L, ULK1, Ki67, or LC3B. Scale bar: 50 μm. **d** Representative images of H&E staining and immunohistochemistry of NEDD4L and ULK1 proteins in pancreatic tumor tissues from KPC mice. Pancreas from KPC mice were dissected at 20 weeks after birth and embedded in paraffin, and pancreas sections were stained with H&E and with antibodies against NEDD4L, ULK1, or Ki67. Scale bars: 50 μm.

To determine the effect of NEDD4L-mediated autophagy inactivation on pancreatic cancer progression, we compared tumor growth in in vivo xenograft mice models administered with human pancreatic cancer cell line MIA PaCa-2 with (shNEDD4L) or without (shCTL) NEDD4L knockdown. As shown in Fig. 6a, tumor growth in mice given shNEDD4L-containing pancreatic cancer xenograft was substantially higher than in mice given shCTL-containing tumors. We next investigated whether autophagy induction mediated by NEDD4L depletion would promote tumor growth in the in vivo mouse model. Daily administration of autophagy inhibitor chloroquine (CQ) via intraperitoneal injection effectively deterred growth in shNEDD4L pancreatic cancer cell-derived tumors, whereas growth of shCTL cell-driven tumors was largely unaffected (Fig. 6b).

We also examined tumor histological phenotypes in xenograft mice using immunohistochemistry (IHC) analysis. Interestingly, the levels of either ULK1 or LC3B increased, as the intensity of NEDD4L staining decreased in the same tumor regions. However, this inverse correlation between NEDD4L and either ULK1 or LC3B levels was not observed in shCTL tumor sections (Fig. 6c). Moreover, the levels of proliferation marker, Ki67 substantially increased in shNEDD4L tumor sections compared to that in shCTL, suggesting that shNEDD4L tumors tend to proliferate faster than shCTL in pancreatic cancer xenograft model (Fig. 6c).

Next, we performed IHC analysis of tumors from xenograft mice driven by either shNEDD4L or shCTL pancreatic cancer cells, with or without CQ injection (from Fig. 6b). As the levels of ULK1 were induced, Ki67 intensities were significantly increased in shNEDD4L cell-driven tumors compared to those in shCTL tumor. However, shNEDD4L tumors from CQ-injected mice, showed significant increase of the levels of Caspase 3, a cell death marker compared to shNEDD4L tumors from vehicle-injected mice, suggesting that autophagy inhibition effectively blocks tumor growth through stimulation of cell death mechanism, particularly in NEDD4L-depleted tumors (Fig. S10). Furthermore, we observed significant inverse correlation between NEDD4L and ULK1 levels in the pancreatic regions from spontaneous pancreatic ductal adenocarcinoma (PDAC) mouse model driven by *KrasLSL-G12D/+*; *Trp53LSL-R172H/+*; *Pdx1-Cre* (KPC). Similar to the results from xenograft mouse, the PDAC tumor regions with higher levels of ULK1 also showed relatively higher levels of Ki67 staining (Fig. 6d). Therefore, these overall results support the critical role of NEDD4L in deregulating the protein stability of ULK1 and ASCT2, thereby effectively diminishing autophagy and further repressing pancreatic cancer growth.

## Discussion

Although diverse mechanisms maintaining ULK1 protein expression have been reported^10,11,13,30^, the molecular mechanisms responsible for ULK1 stability have not yet been fully elucidated.

Herein, we investigated the mechanisms by which ULK1 is modulated by an E3 ubiquitin ligase, NEDD4L, and suggest the physiological significance of these molecular regulations on tumor progression. We propose a novel mechanism showing that cancers expressing low levels of NEDD4L enhance autophagy and mitochondrial metabolism via deregulating the protein activity of ULK1 and a glutamine transporter, and so promote tumor development. Our findings in this study that when NEDD4L is suppressed, certain cancer cells appear to have more intact and functional mitochondria compared to counterpart cells with wild-type levels of NEDD4L, implying that NEDD4L might have an inhibitory role in maintaining mitochondrial metabolic functionality.

First, we showed that ULK1 associates with NEDD4L in pancreatic cancer cells. Ubiquitination of ULK1 decreased when NEDD4L expression was suppressed, resulting in higher levels of ULK1 and increased autophagy activity. These results indicate that NEDD4L suppresses autophagy by reducing cellular ULK1 protein levels. In addition, NEDD4L influenced the stability of glutamine transporters. The protein levels of ASCT2 were markedly decreased under nutrient depletion; however, the downregulation of these proteins was substantially less pronounced when NEDD4L expression was suppressed. Autophagy activity enhanced by NEDD4L depletion was repressed by the knockdown of identified NEDD4L-target genes—either ULK1 or ASCT2—suggesting that the stabilization of ULK1 or ASCT2 mediated by low expression of NEDD4L is critical for activating autophagy.

Moreover, we found that NEDD4L inversely regulated mitochondrial energy metabolism by affecting the OCR, MMP, and mitochondrial morphology; furthermore, such effect of NEDD4L on mitochondrial integrity was compromised by the additional knockdown of either ULK1 or ASCT2. We could therefore conclude that NEDD4L depletion in cancer cells facilitated ULK1-mediated autophagy and ASCT2-mediated glutamine uptake to supply appropriate fuel to activate mitochondrial metabolism and maintain mitochondrial functional integrity. Cancers with low NEDD4L expression tend to display enhanced mitochondrial metabolic functions due to ULK1-mediated autophagy. Therefore, the increased mitochondrial energy production found in NEDD4L-depleted cancers can be effectively targeted by autophagy inhibition, which can be considered as a promising therapeutic approach.

Based on these unique features of NEDD4L, we were prompted to next investigate the effect of NEDD4L expression on cancer cell proliferation and survival. Low-NEDD4L expression improved cancer cell growth and viability. Moreover, NEDD4L-depleted cells were sensitized to nutrient deprivation with the knockdown of either ULK1 or ASCT2. Similarly, shNEDD4L cells were sensitized to pharmacological inhibition of ULK1 or ASCT2, as well as to the blockade of mitochondrial OXPHOS when compared to shCTL cells, suggesting that NEDD4L-knockdown cancer cells rely on mitochondrial metabolism to sustain cell growth and survival. Finally, in vivo tumor assessment showed that shNEDD4L cells promote tumor progression to a greater extent than shCTL cells in xenograft pancreatic cancer mouse model, the enhanced tumor growth was then repressed by treatment with the autophagy inhibitor CQ. More interestingly, the protein levels of NEDD4L and ULK1 or ASCT2 were inversely expressed in both xenograft tumor and tumor from spontaneous PDAC mouse model, KPC mice. Overall, the results suggest that NEDD4L plays a key role in suppressing tumor progression by inhibiting autophagy and mitochondrial respiratory function. Our study also suggests the importance of the regulatory mechanisms between the ubiquitin proteasome system and autophagy pathway, which further links cancer-specific metabolic alterations to the response metabolic stress, and potentially for certain types of tumorigenesis.

Multiple cancer cell types have been previously observed to have lowered expression of NEDD4L compared to healthy counterparts^23,24,31,32^. In contrast, certain types of cancer, such as melanomas, express relatively high levels of NEDD4L, and tumor growth is inhibited when NEDD4L expression is suppressed^27^. Moreover, the expression of various oncogenic effector proteins can be regulated by HECT domain-containing E3 ubiquitin ligases including NEDD4L. Due to these conflicting findings, the role of NEDD4L during tumor development has not yet been fully defined.

The Cancer Genome Atlas (TCGA) datasets (TCGA 2012 provisional; *cbioportal.org*) showed that NEDD4L levels were downregulated in multiple human tumor patients, and were inversely correlated with clinical outcome. In human pancreatic cancers, more than 15% of Cancer Cell Line Encyclopedia (CCLE; gepia.cn) datasets showed the down-regulation of *Nedd4l* gene expression. Moreover, patient groups with altered NEDD4L expression showed a relatively shorter overall survival rate in pancreatic cancer patients, although this was not statistically significant (https://www.oncomine.org/).

In summary, our results suggest that the E3 ubiquitin ligase NEDD4L downregulates ULK1 and ASCT2 protein levels, thereby reducing autophagy activity and mitochondrial metabolic activity. Specifically, pancreatic cancer cells with low levels of NEDD4L predominantly relied on the activation of autophagy and mitochondrial OXPHOS for cancer growth and survival in both in vitro and in vivo studies. Our results revealed a possible mechanism by which NEDD4L, as a tumor suppressor, prohibits autophagy activity under metabolic stress conditions via the destabilization of an autophagy protein, ULK1, and a glutamine transporter, ASCT2, thereby inhibiting mitochondrial functionality and ultimately suppressing tumor progression. These findings suggest a novel therapeutic strategy for patients with low levels of NEDD4L in cancer.

## Materials and methods

### Cell lines

HEK293T, HeLa, and MIA PaCa-2 cells were purchased from the American Type Culture Collection (ATCC; Manassas, VA, USA). All cells were maintained at 5% CO_2_ and 37 °C in Dulbecco’s modified Eagle’s medium (DMEM) supplemented with 10% fetal bovine serum (FBS; Hyclone, Logan, UT, USA), 100 U/mL penicillin, and 100 μg/mL streptomycin (Gibco, Waltham, MA, USA). DMEM without glutamine or glucose (Gibco) was supplemented with 10% FBS (Hyclone, Logan, UT, USA). EBSS (Hyclone) was used for nutrient-deprived condition. For amino acid starvation medium, Hank’s balanced saline solution (HBSS) was supplemented with 10% dialyzed FBS, glucose, vitamins, HEPES, and minerals at the same concentrations as in DMEM.

GFP-LC3 or mCherry- GFP-LC3 was stably expressed in HeLa and MIA PaCa-2 cells using a retroviral vector following standard protocols for viral transduction. To knockdown NEDD4L, cells were transduced with the shNEDD4-2 lentiviral vector (Addgene #27016), and control cells were transfected with a scramble shRNA control lentivirus.

### Antibodies and reagents

Primary antibodies against ULK1 (8054), pULK1 (5869), ASCT2 (8057), COXIV(4850), and LC3B (2775), Caspase-3(9661) were purchased from Cell Signaling Technology (Danvers, MA, USA); those against NEDD4L (A302–514A), β-actin (A300–491A), and HA (A190–108A) were purchased from Bethyl Laboratories (Montgomery, TX, USA); those against Atg13 (gtx122766) were purchased from GeneTex (Irvine, CA, USA); those against FLAG M2 (F1804) and FLAG (F7425) were purchased from Sigma Aldrich (St. Louis, MO, USA); and those against TOM20 (SC-11415), BECN1(SC-11427), Ub(SC-8017), and β-actin (SC47778) were purchased from Santa Cruz Biotechnology (Dallas, TX, USA). The total OXPHOS Human WB antibody cocktail (ab110411) was purchased from Abcam (Cambridge, UK). The secondary antibodies, horseradish peroxidase-linked anti-rabbit (A120–101P) and anti-mouse (A90–116P), were purchased from Bethyl Laboratories.

For IHC, primary antibodies against NEDD4L (IHC-00713; Bethyl Laboratories); those against ULK1 (ab128859) and Ki67 (ab15580) were purchased from Abcam (Cambridge, UK) and those against LC3B (2775), Caspase-3(9661) were purchased from Cell Signaling Technology.

CQ (C6628), MG132 (M7449), GPNA (l-Glutamic acid γ-(p-nitroanilide) hydrochloride, G6133), CCCP (carbonyl cyanide 3-chlorophenylhydrazone, C2759), Rotenone (R8875), Antimycin A from Streptomyces (A8674), and phosphatase inhibitor cocktails 2 and 3 were purchased from Sigma Aldrich. Protease inhibitor cocktail tablets were purchased from Roche Applied Bioscience (Penzberg, Germany). SBI-0206965(S7885) was purchased from Selleck Chemicals (Houston, TX, USA). DAPI (D3571), Lipofectamine 2000 (11668019), RNAi Max (13778150), MitoTracker Green (M7514) and JC-1 (T3168) were purchased from Thermo Fisher Scientific (Waltham, MA, USA). Oligomycin (sc-203342) was purchased from Santa Cruz Biotechnology (Dallas, TX, USA).

### DNA construct and siRNA

For the construction of the HA-NEDD4L or NEDD4L, a polymerase chain reaction (PCR)-amplified DNA fragment encoding NEDD4L was inserted between the *NotI* and *XbaI* sites of the pcDNA3 or pcDNA3.1 vector (Invitrogen). A PCR-amplified DNA fragment encoding ASCT2 was inserted between the *EcoRI* sites of the pcDNA3.1–3×Flag vector. The full-length NEDD4L and ASCT2 cDNA were provided by the Korea Human Gene Bank. Plasmids for shRNA NEDD4L (#27016) were kindly provided by Dr. Joan Massague (Memorial Sloan-Kettering Cancer Center, New York, NY, USA) through Addgene (Watertown, MA, USA). A plasmid encoding GFP-LC3 in a MigRI-based retroviral vector was generously provided by Dr. Craig Thompson (Memorial Sloan-Kettering Cancer Center). The pcDNA3-HA, plasmids encoding HA-ULK1 and HA-ubiquitin were provided by Dr. Seok Hee Park (Sungkyunkwan University, Seoul, Korea). siRNA targeting the respective genes of interest and negative control siRNA (non-targeting pool) were purchased from Genolution Inc (Seoul, Korea).

The following siRNA sequences were used for the indicated target genes: siULK1, 5′GUGGCCCUGUACGACUUCCAGGAAA-3′ (#1) and 5′GCACAGAGACCGTGGGCAA-3′ (#2); siASCT2, 5′-UGAUACAAGUGAAGAGUGA-3′; and siNEDD4L, 5′AACCACAACACAAAGUCACAG-3′.

### Immunoprecipitation

ULK1 was tagged with a FLAG or HA epitope. NEDD4L and ubiquitin were tagged with a human influenza HA epitope. Epitope-tagged proteins were co-expressed in HEK293T cells. HEK293T cells were rinsed in ice-cold phosphate-buffered saline and lysed in lysis buffer composed of 1% NP-40, 20 mM Tris-HCl, 150 mM NaCl, 10% glycerol, 2 mM EDTA, 10 mM NaF, 1 mM Na_3_O_4_V, 0.2 mM PMSF, and including a protease inhibitor cocktail (11836153001; Roche Applied Bioscience) and 1% phosphatase inhibitor cocktail (Sigma Aldrich). Next, 0.5 mg lysates for co-immunoprecipitation in cells overexpressing the above proteins were incubated with 2 μg primary antibodies for anti-FLAG M2 (Sigma Aldrich), anti-HA antibody (Bethyl Laboratories), or rabbit IgG (Sigma Aldrich) at 4 °C with overnight shaking after adding 50 μl protein A agarose beads (GenDEPOT, Katy, TX, USA). Immunoprecipitates were washed three times with wash buffer and then eluted by boiling in sodium dodecyl sulfate (SDS) sample buffer with β-mercaptoethanol (β-ME) for 5 min. Then, the immunoprecipitate complex was analyzed by liquid chromatography mass spectrometry (LC–MS) or immunoblotted with the indicated antibodies.

### Ni-NTA pull-down assay

Ni-NTA pull-down assay was performed with His-Ubiquitin as described previously^33^. Briefly, HEK293 cells expressing HA-ULK1 and His-Ubiquitin were lysed in 6 M guanidine-HCl buffer (pH 8.0). His-ubiquitin-conjugated proteins were purified by incubation with Ni^2+^-NTA agarose beads (Qiagen), washed with 25 mM Tris, 20 mM imidazole washing buffer, eluted in sample buffer, and then analyzed by immunoblotting.

### LC–MS/MS analysis

The protein samples were precipitated using cold acetone, reduced with 10 mM dithiothreitol (DTT), and alkylated with iodoacetamide (IAA). The alkylated samples were digested with mass spec grade trypsin/lys-C mix in 50 mM Tris-HCl (pH 8) for 12 h at 37 °C. The digested peptides were analyzed by a Q Exactive hybrid quadrupole-orbitrap mass spectrometer (Thermo Fisher Scientific) coupled with an Ultimate 3000 RSLCnano system (Thermo Fisher Scientific). The peptides were loaded onto trap columns (100 μm × 2 cm) packed with Acclaim PepMap100 C18 resin, separated on an analytical column (EASY-Spray column, 75 μm × 50 cm, Thermo Fisher Scientific), and sprayed into the nano-electrospray ionization source. The Q Exactive Orbitrap mass analyzer was operated in a top ten data-dependent method. Full MS scans were acquired over a range of 300–2000 *m*/*z* with a mass resolution of 70,000 (at 200 *m*/*z*). The automatic gain control target value was 1.0 × 10^6^. The ten most intense peaks with charge state ≥2 were fragmented in the higher-energy collisional dissociation collision cell with normalized collision energy of 30, and tandem mass spectra were acquired in the Orbitrap mass analyzer with a mass resolution of 17,500 at 200 *m*/*z*.

Database searching of all raw data files was performed using Proteome Discoverer 2.2 software (Thermo Fisher Scientific). SEQUEST-HT was used for database searching against the Swiss-Prot *Homo sapiens* database. Database searching against the corresponding reversed database was also performed to evaluate the false-discovery rate (FDR) of peptide identification. The database searching parameters included precursor ion mass tolerance 10 ppm, fragment ion mass tolerance 0.08 Da, fixed modification for carbamidomethyl cysteine, and variable modifications for methionine oxidation. We obtained an FDR of less than 1% on the peptide level and filtered for high peptide confidence.

### Fluorescence microscopy analysis of autophagy

Cell lines stably expressing LC3B tagged with GFP were used for monitoring autophagy activity by confocal fluorescence microscopy. Cells stably expressing the tandem mCherry-GFP-LC3 construct were also used. MIA PaCa-2 and HeLa cells stably expressing GFP-LC3 or mCherry-GFP-LC3 and transfected with either control or NEDD4L shRNA were cultured in a glass-bottomed chamber (LabTek; Thermo Fisher Scientific) overnight, and then replaced with DMEM culture medium containing the indicated chemicals or starvation media for the indicated time periods. Nuclei were stained using DAPI or Hoescht-33342. Images were acquired with the LSM780 confocal fluorescent microscope (Carl Zeiss, Oberkochen, Germany) and the percent of either GFP-LC3 puncta area or mCherry-LC3 puncta area were normalized to the DAPI-stained area, which was quantified using ZEN black software (Carl Zeiss). The area of LC3 puncta was counted in five different arbitrary areas from three independent experiments.

GFP-LC3 puncta levels were also measured using the image-based HCS system Operetta CLS (PerkinElmer, Waltham, MA, USA) and quantified as the percent of GFP-LC3 puncta area normalized to cell area using Harmony software (PerkinElmer).

### Fluorescence microscopy analysis of subcellular mitochondrial morphology

MIA PaCa-2 cells and HeLa cells transfected with siCTL or siNEDD4L were cultured in a glass-bottomed chamber with DMEM medium. At the indicated time points, cells or nuclei were stained with DAPI or Hoescht-33342, respectively, and mitochondria were stained with MitoTracker Green (Thermo Fisher Scientific) according to the manufacturer’s instructions. Then, cells were further incubated with either vehicle or 10 µM CCCP for 1 h to induce the loss of mitochondrial integrity. Images were captured with an LSM880 confocal fluorescent microscope with AiryScan (Carl Zeiss) at ×1000 magnification or higher, and then quantified with ZEN black software (Carl Zeiss). At least five distinct regions were imaged per sample from three independent experiments and the average area of MitoTracker Green signals were normalized to the nucleus area.

### Glutamine measurement

Glutamine consumption was measured using the BioProfile analyzer (Nova Biomedical, MA, USA). Briefly, cells were plated in 6-well plates in complete medium, which was replaced by DMEM media freshly, the following day and then incubated for another 24 h. Glutamine concentration in the media was then measured and normalized to the number of cells in each well. The controls were plated with an equal volume of medium but no cells, incubated identically as the cell-containing plates. Glucose consumption and lactate production rate were measured similarly using the BioProfile analyzer.

### Cell proliferation and death assay

Cell proliferation was measured using the image-based cell proliferation analyzer IncuCyte^TM^ (Essen Instruments, Ann Arbor, MI, USA). Cells were cultured in nutrient-complete DMEM media on multi-well plates overnight and imaged throughout the indicated time period. IncuCyte^TM^ automated cell proliferation detector was used to measure cell proliferation through quantitative kinetic processing metrics derived from time-lapse image acquisition and presented as a percentage of cell confluence over time. For cell proliferation experiments with overexpression, cells were ectopically transfected with HA-tagged NEDD4L and subsequently measured their proliferation.

Cell viability was determined by annexin V and PI staining following standard protocols at the indicated time periods (556547, BD Biosciences, San Jose, CA, USA). Cells negative for both annexin V and PI were considered live cells. The proportion of dead cells was measured based on the number of annexin V and PI single and both-stained cells. The fluorescence of stained cells was detected using the LSR-Fortessa FACS analyzer (BD Biosciences).

### Flow cytometry analysis for measurement of MMP

JC-1 (Thermo Fischer Scientific) was used according to the manufacturer’s instructions to measure changes in the MMP of cells. Briefly, JC-1 (5,5′,6,6′-tetrachloro-1,1′,3,3′-tetraethylbenzimi-dazolylcarbocyanine iodide) is a lipophilic cationic probe that shows changes in the MMP. In mitochondria with a disrupted membrane potential, the dye’s fluorescence in its monomeric form can be detected by fluorescein isothiocyanate (FITC). Cells were cultured in nutrient-complete media and then treated with 10 µM CCCP or 1 µM/3 µM Oligomycin/Antimycin A (OA) for 2 h prior to staining with JC-1 reagent for 30 min at 37 °C and 5% CO_2._ The MMP was assessed based on the changes in phycoerythrin (PE) and FITC signals for JC-1 in the LSR-Fortessa FACS analyzer (BD Biosciences).

### Western blotting

Cells were harvested in ice-cold RIPA lysis buffer (50 mM Tris-Cl, pH 7.4, 150 mM NaCl, 1% NP-40, 0.5% Na-deoxycholate, 0.1% SDS, 1 mM EDTA) containing protease inhibitor cocktail (Roche Applied Bioscience) and phosphatase inhibitor (Sigma Aldrich). Soluble lysate fractions were isolated by centrifugation at 20,000 × *g*, for 20 min at 4 °C and quantified using the Pierce bicinchoninic acid (BCA) Protein Assay kit (Thermo Fisher Scientific). Samples were resolved by SDS polyacrylamide gel electrophoresis using equal concentrations of protein and transferred to polyvinylidene fluoride membranes. The membranes were blocked with 5% skim milk and then probed with the indicated primary and secondary antibodies following standard protocols.

### Cellular energy metabolism analysis

The cellular OCR was measured using the Seahorse XFe96 extracellular flux analyzer (Agilent Technologies, Santa Clara, CA, USA). Cells were plated in appropriate multi-well plates and then the cartridge filled with calibrant buffer (XF calibrant, 100840-000; Agilent Technologies) were set up on the cultured cells (20,000 cell/well/96 well plate). All buffers were prepared with XF base medium (10252-100; Agilent Technologies) and adjusted to reflect the pH of the cell culture medium. For real-time measurements in living cells, the Mito-stress test kit (MST:103015-100; Agilent Technologies) was used according to the manufacturer’s instructions at a final concentration of 1 μM Oligomycin, 0.5 μM FCCP, and 0.45 μM Rotenone/Antimycin A, respectively, during measurement. The levels of OCR in real time were normalized by cell numbers which were quantified by image-based HCS system Operetta CLS, immediately after the analysis.

### In vivo tumor assessment

Six-week-old female BALB/C nude mice (Orient Bio Inc., Seongnam, Korea) were handled using aseptic procedures and allowed to adjust to local conditions for 1 week before experimental manipulations began. MIA PaCa-2 shCTL and shNEDD4L cells (5 × 10^6^) were mixed at a 1:1 dilution with Matrigel (354234; Corning Inc., Corning, NY, USA) and injected subcutaneously into both flanks of each mouse at a total final volume of 100 μL. Each group has five mice. When tumors reached an average volume of 100 mm^3^, mice were treated with CQ (25 mg kg^−1^ day^−1^), daily via intraperitoneal injection for up to 4 weeks. Tumor growth was evaluated by the measurement of two perpendicular diameters of the tumors and tumor size was calculated using the formula 4*π*/3 × (width/2)^2^ × (length/2). The tumors were harvested at the experimental endpoint. Animal experiments were performed in accordance with the protocols approved by the Institutional Animal Care and Use Committee at the National Cancer Center, Republic of Korea. The methods applied in this study were performed in accordance with the approved guidelines.

### Mouse breeding

KC mouse model was received from NCI mouse repository (NCI, Bethesda, MD, USA). The mice were used for generate KPC (*KrasLSL-G12D/+*; *Trp53LSL-R172H/+*; *Pdx1-Cre*) mouse by routine breeding. The KPC model of PDAC was first described in 2005 and incorporates, through Cre-Lox technology, the conditional activation of mutant endogenous alleles of the Kras and Trp53 genes^34^

### PDAC mouse and pancreas isolation

*Pdx1-Cre*, *LSL-Kras*^*G12D*^, and *LSL-Trp53*^*R172H*^ mice were received from NCI mouse repository (http://mouse.ncifcrf.gov). *LSL-Kras*^*G12D*^; *Trp53* ^*R172H*^; *Pdx1-Cre* (KPC) mice were obtained by crossing these mice. These mice were maintained in C57BL/6J genetic background. Cre-mediated recombination in the KPC mouse model leads to conditional expression of mutant Kras and Trp53 gene specifically in the mouse pancreas. Because this model has the key features of human PDAC, it is the most extensively studies genetic model of PDAC in cancer research and preclinical studies. Pancreas was isolated from 22-month-old KPC mouse. This study was reviewed and approved by the Institutional Animal Care and Use Committee (IACUC) of the National Cancer Center Research Institute, which is an Association for Assessment and Accreditation of Laboratory Animal Care International (AAALAC International) accredited facility that abides by the Institute of Laboratory Animal Resources guide (protocols: NCC-17–391; NCC-15–239)

### Immunohistochemistry

For xenograft samples and pancreatic tissue from KPC mice, dissected tissues were fixed immediately after removal in 10% buffered formalin solution for a maximum of 24 h at room temperature before being dehydrated and paraffin-embedded under a vacuum. The tissue sections were deparaffinized with EZ Prep buffer (Ventana Medical Systems, Santa Clara, USA). Antigen retrieval was performed with CC1 buffer (Ventana Medical Systems), and sections were blocked for 30 min with Background Buster solution (Innovex, Lincoln, RI, USA). IHC detection was performed using the Discovery XT processor (Ventana Medical Systems). All tumor tissues were harvested from mice and fixed in 4% PFA overnight. Fixed tissues were dehydrated, embedded in paraffin, and sliced into 3-µm sections. The tissue sections were deparaffinized with EZ Prep buffer, and antigen retrieval was performed with CC1 buffer and heat treatment in citrate buffer at pH 6.0 (Ribo CC, Ventana Medical Systems). Tumor sections were incubated with the indicated primary antibodies and detection was performed with a DAB detection kit (Ventana Medical Systems) according to the manufacturer’s instructions, followed by counterstaining with hematoxylin (Ventana Medical Systems). Images were obtained using Vectra Polaris (PerkinElmer).

### Statistical analyses

Immunoblotted proteins or IHC DAB intensities were quantified and using ImageJ software (NIH, Bethesda, MD, USA) and normalized by loading control or nucleus area. Data are expressed as the mean ± standard deviation or standard error of the mean which are from at least three independent experiments. Statistical significance was calculated using Student’s *t* test in Graph Pad Prism 8. A value of *P* < 0.05 was considered statistically significant (*P < 0.05; **P < 0.01).

## Supplementary information

Supplementary Table 1

Supplementary Figure Legends

Supplementary Figure 1

Supplementary Figure 2

Supplementary Figure 3

Supplementary Figure 4

Supplementary Figure 5

Supplementary Figure 6

Supplementary Figure 7

Supplementary Figure 8

Supplementary Figure 9

Supplementary Figure 10
