# Supplementary material for: NEDD4L downregulates autophagy and cell growth by modulating ULK1 and a glutamine transporter
Source: Cell Death Dis. 2020 Jan 20;11(1):38. doi: 10.1038/s41419-020-2242-5 (PMC6971022; doi:10.1038/s41419-020-2242-5)
Supplement: Supplementary file 1 — Supplementary Table 1 [file 41419_2020_2242_MOESM1_ESM.docx]

| **Gene symbol** | **Accession** | **Description** | **Score** | **Coverage(%)** | **Peptide** |
| --- | --- | --- | --- | --- | --- |
| *pkm2* | P52480 | Pyruvate kinase PKM | 5.81 | 6.03 | 2 |
| *nedd4l-2* | G3X9H8 | E3 ubiquitin-protein ligase | 5.80 | 1.99 | 5 |
| *vimentin* | P20152 | Vimentin | 4.58 | 7.08 | 2 |
| *map4* | E9PWC0 | Microtubule-associated protein | 2.64 | 2.77 | 8 |
| *ldha* | D3Z736 | L-lactate dehydrogenase | 2.23 | 6.33 | 4 |

**Supplementary table1**
