# Supplementary material for: NEDD4L downregulates autophagy and cell growth by modulating ULK1 and a glutamine transporter
Source: Cell Death Dis. 2020 Jan 20;11(1):38. doi: 10.1038/s41419-020-2242-5 (PMC6971022; doi:10.1038/s41419-020-2242-5)
Supplement: Supplementary file 2 — Supplementary Figure Legends [file 41419_2020_2242_MOESM2_ESM.docx]

**SUPPLEMENTARY FIGURE LEGENDS**

***Supplementary Table 1.*** Mouse embryonic fibroblast (MEF) cells were transiently transfected with vector alone or FLAG-ULK1 encoding plasmid, respectively. After 24h, cell lysates were immunoprecipitated with anti-FLAG antibody (M2) or with IgG as a negative control ULK1-interacting proteins were eluted from the immuno-complex and identified by LC-MS/MS analysis. The top lists of ULK1 interacting partners are given.

***Supplementary Figure 1.*** Cell lysates were prepared from Panc-1 stably expressing shCTL or shNEDD4L and immunoblotted against ULK1 and NEDD4L. β-actin was used as loading control. Error bars indicate the mean ± SEM for three independent experiments. ** *P* < 0.01

***Supplementary Figure 2.*** shCTL and shNEDD4L MIA PaCa-2 cells were plated overnight and collected for immunoblot analysis. Lysates were resolved on SDS-PAGE and immunoblotted against Atg13, Beclin1, and NEDD4L. β-actin was used as loading control.

***Supplementary Figure 3.*** shCTL and shNEDD4L MIA PaCa-2 cells were plated overnight and subsequently starved in EBSS media. Cells were collected at indicated time points and lysates were immunoblotted with antibody against LC3. β-actin was used as loading control. Error bars indicate the mean ± SEM for three independent experiments.

***Supplementary Figure 4.*** Panc-1 and PA-TU 8988T cells stably expressing shCTL and shNEDD4L were plated overnight and subsequently treated with vehicle (DMSO) or Oligomycin/Antimycin A (OA; 1 μM/3 μM) for 2h to induce mitochondrial dysfunction. Cells were then stained with JC-1 mitochondrial dye and analyzed by flow cytometry. Error bars indicate the mean ± SEM for three independent experiments.

***Supplementary Figure 5.*** shCTL and shNEDD4L Panc-1 cells were treated with vehicle (DMSO), CCCP (10 μM), or Oligomycin/Antimycin A (OA; 1 μM/3 μM) for 4h. Cell lysates were immunoblotted against COXIV, TOM20, and a cocktail antibody of mitochondrial OXPHOS complex. Error bars indicate the mean ± SEM for three independent experiments. * *P* < 0.05

***Supplementary Figure 6.*** shCTL and shNEDD4L MIA PaCa-2 and Panc-1 cells were plated overnight. After 24h, the culture media was collected and cells enumerated. Glucose and lactate levels in the collected media in comparison to fresh culture media were measured using a metabolite analyzer. Metabolic rates of glucose uptake and lactate production were derived after normalization by the number of cells. Error bars indicate the mean ± SEM for three independent experiments.

***Supplementary Figure 7.*** shCTL and shNEDD4L PA-TU 8988T cells were plated overnight and subsequently starved in EBSS media. Cells were collected at indicated time points and lysates were immunoblotted with antibodies against ULK1 and ASCT2. β-actin was used as loading control. Error bars indicate the mean ± SEM for three independent experiments. * *P* < 0.05; ** *P* < 0.01.

***Supplementary Figure 8.*** shCTL and shNEDD4L MIA PaCa-2 cells were reverse-transfected with siCTL, siULK1, siASCT2 for 48hr. Subsequently, cells treated with vehicle (DMSO) or CCCP (10μM) for 2h to induce mitochondrial dysfunction, and then cells were stained with JC-1 mitochondrial dye and analyzed by flow cytometry. Error bars indicate the mean ± SEM for three independent experiments. * *P* < 0.05; ** *P* < 0.01.

***Supplementary Figure 9.*** shCTL or shNEDD4L MIA PaCa-2, PA-TU 8988T and BxPC3cells or MIA PaCa-2, PA-TU 8988T after ectopic expression of NEDD4L were incubated in the IncuCyteTM analyzer to monitor cell proliferation, respectively. Cell confluence levels were measured real-time and presented as a percentage using the IncuCyte^TM^ analyzer. Error bars indicate the mean ± SEM for three independent experiments. * *P* < 0.05; ** *P* < 0.01.

***Supplementary Figure 10.*** Immunohistochemistry of NEDD4L and ULK1 proteins detected from tumor tissues in Fig 6B. Tumors derived from shCTL or shNEDD4L MIA PaCa-2 cells either with CQ injection or with vehicle injection were dissected after tumor assessment test and embedded in paraffin, and tumor sections were stained with antibodies against NEDD4L, ULK1, Ki67,Caspase-3 or LC3B Scale bar: 50 μm. The levels of DAB for Ki67 or Caspase-3 were quantified by Image J and error bars indicate the mean ± SEM for three independent experiments. * *P* < 0.05; ** *P* < 0.01.
