# Supplementary figures and images for: NEDD4L downregulates autophagy and cell growth by modulating ULK1 and a glutamine transporter
Source: Cell Death Dis. 2020 Jan 20;11(1):38. doi: 10.1038/s41419-020-2242-5 (PMC6971022; doi:10.1038/s41419-020-2242-5)

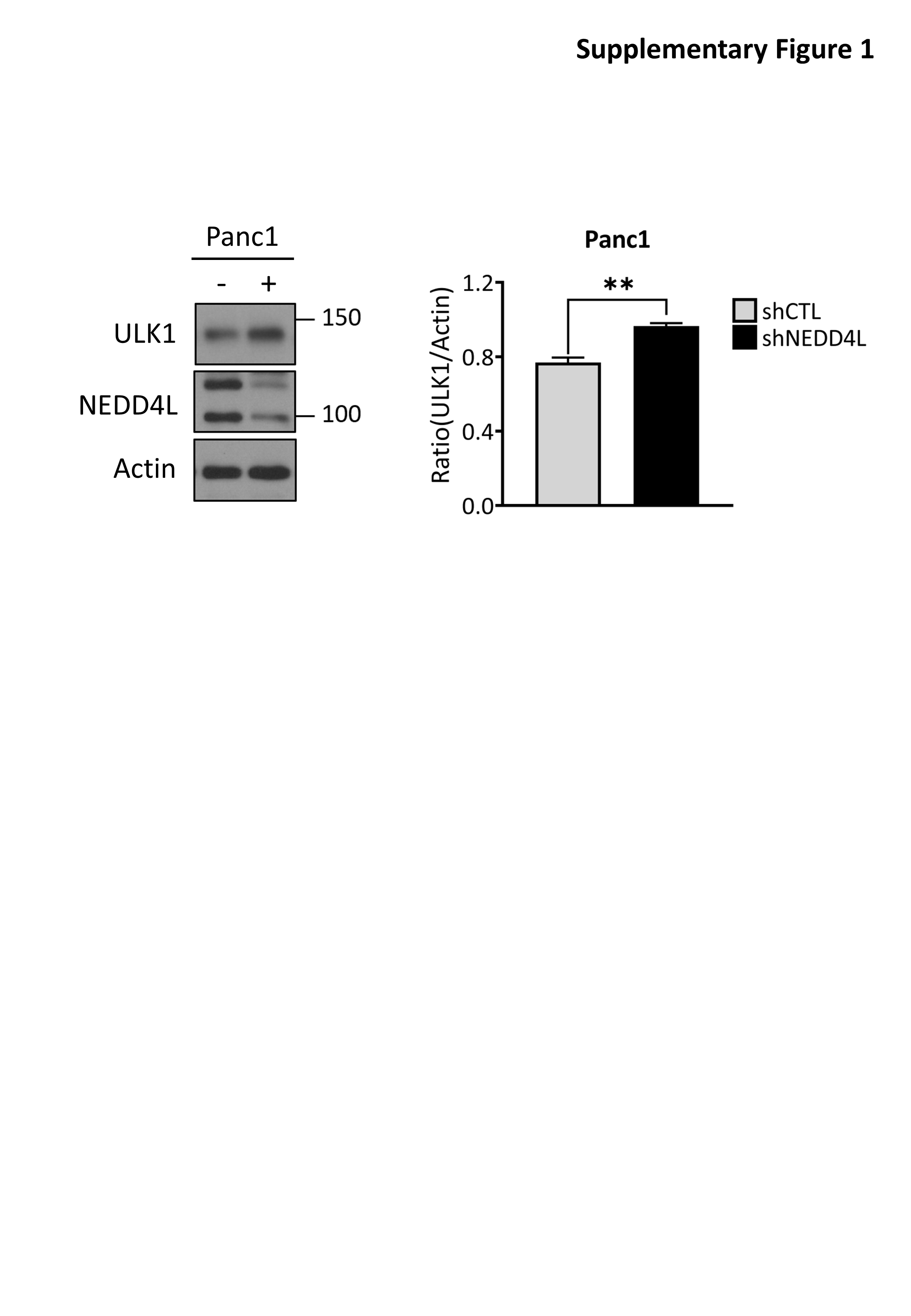

Supplement: Supplementary file 3 — Supplementary Figure 1 [file 41419_2020_2242_MOESM3_ESM.tif]

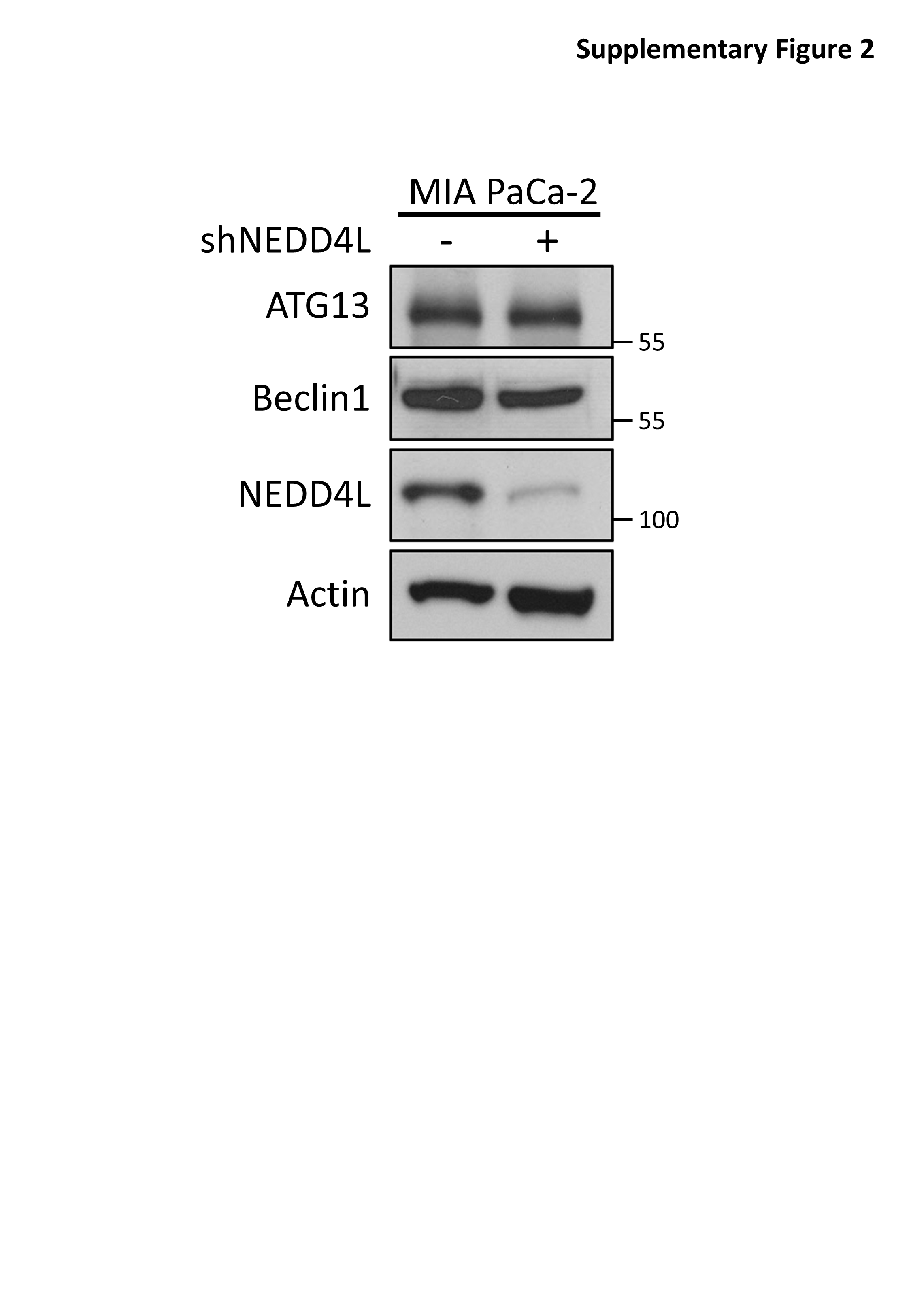

Supplement: Supplementary file 4 — Supplementary Figure 2 [file 41419_2020_2242_MOESM4_ESM.tif]

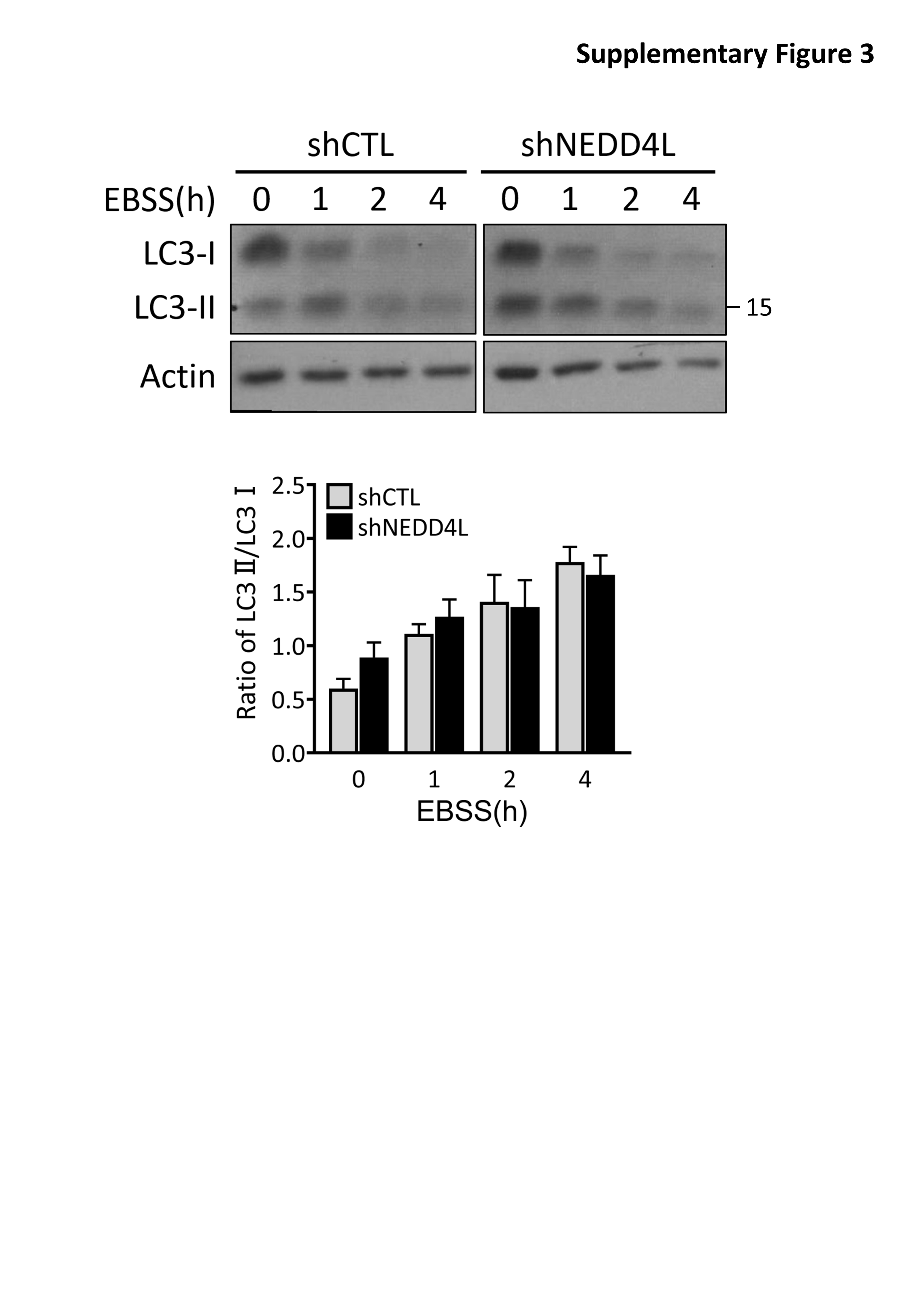

Supplement: Supplementary file 5 — Supplementary Figure 3 [file 41419_2020_2242_MOESM5_ESM.tif]

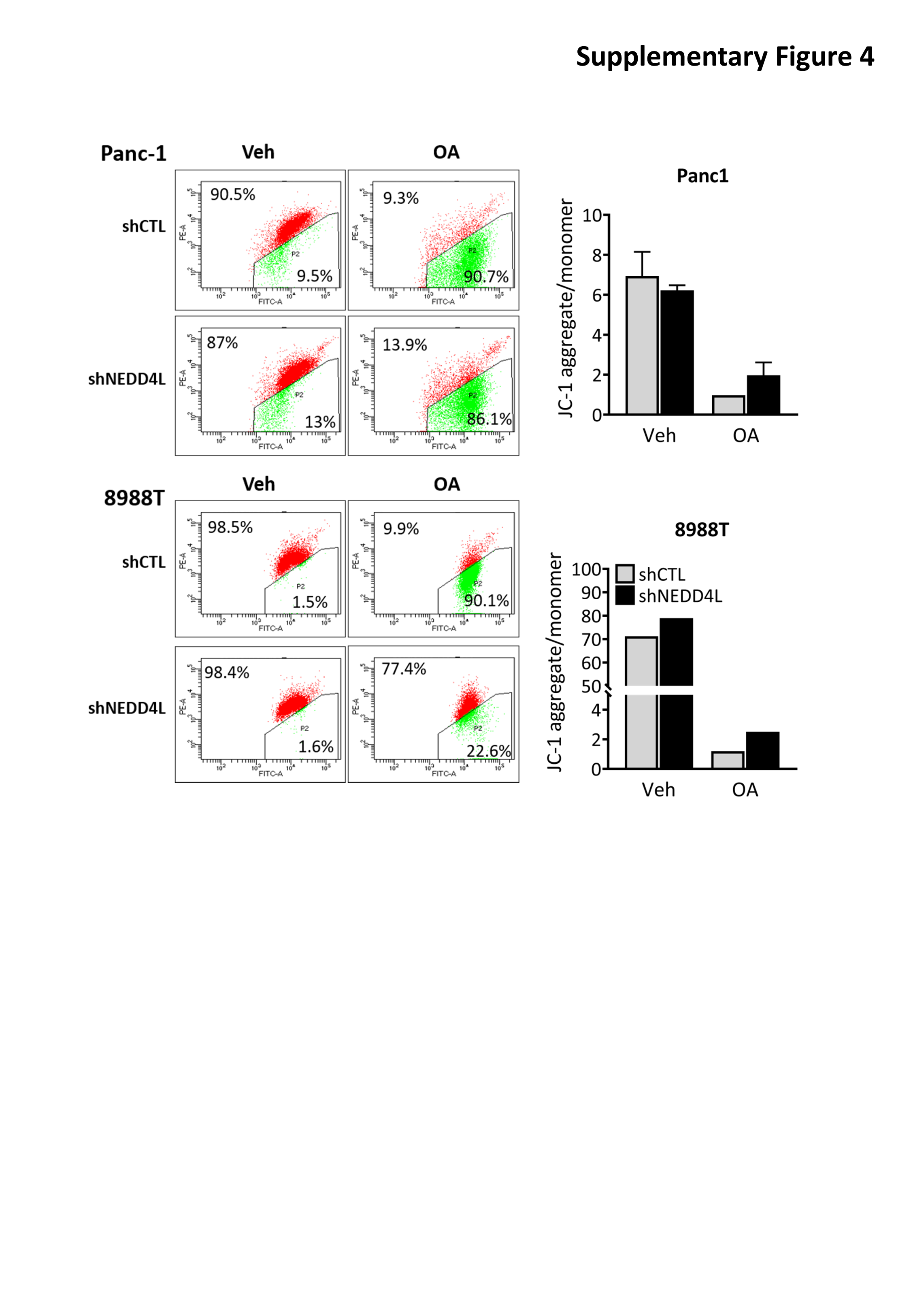

Supplement: Supplementary file 6 — Supplementary Figure 4 [file 41419_2020_2242_MOESM6_ESM.tif]

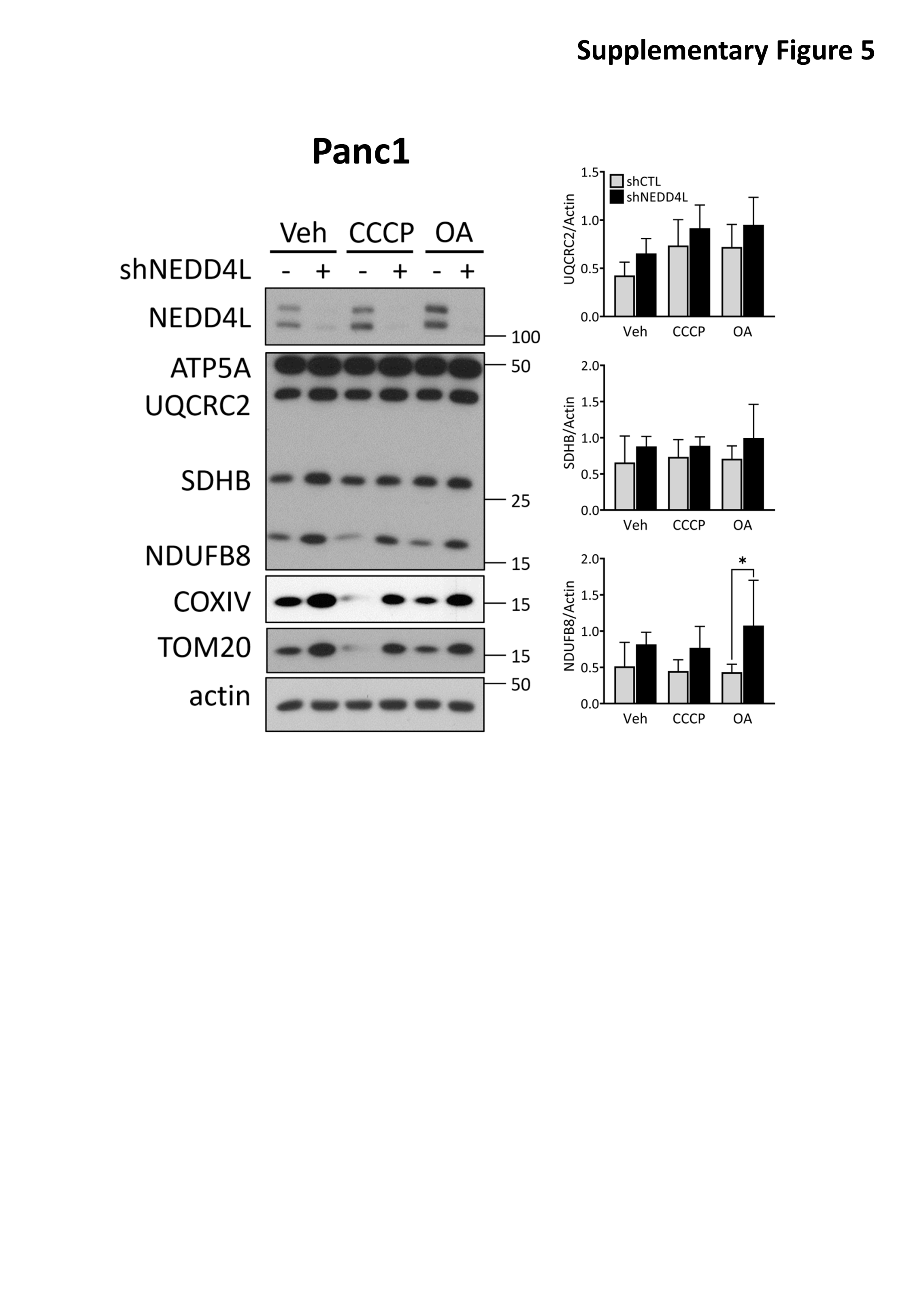

Supplement: Supplementary file 7 — Supplementary Figure 5 [file 41419_2020_2242_MOESM7_ESM.tif]

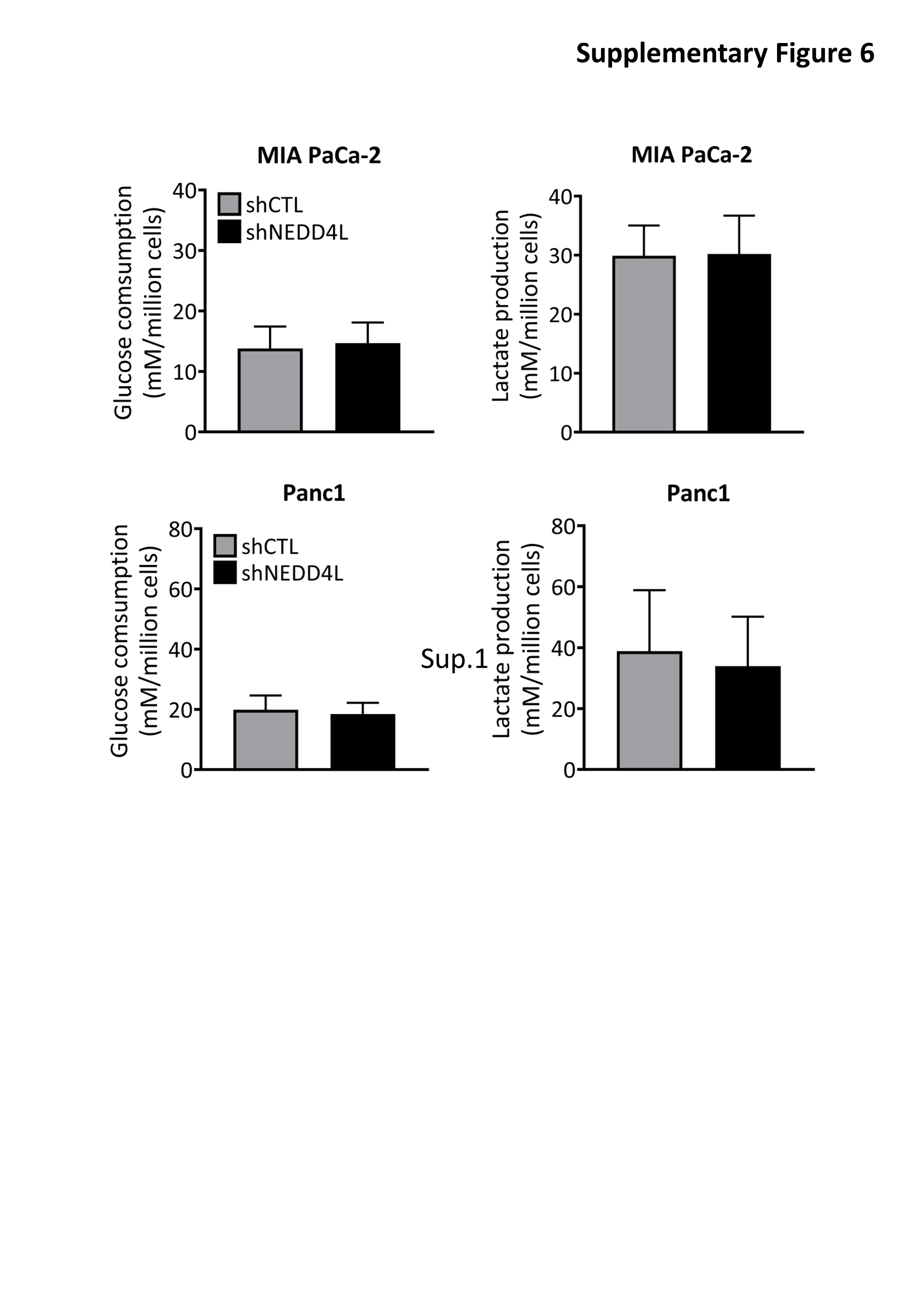

Supplement: Supplementary file 8 — Supplementary Figure 6 [file 41419_2020_2242_MOESM8_ESM.tif]

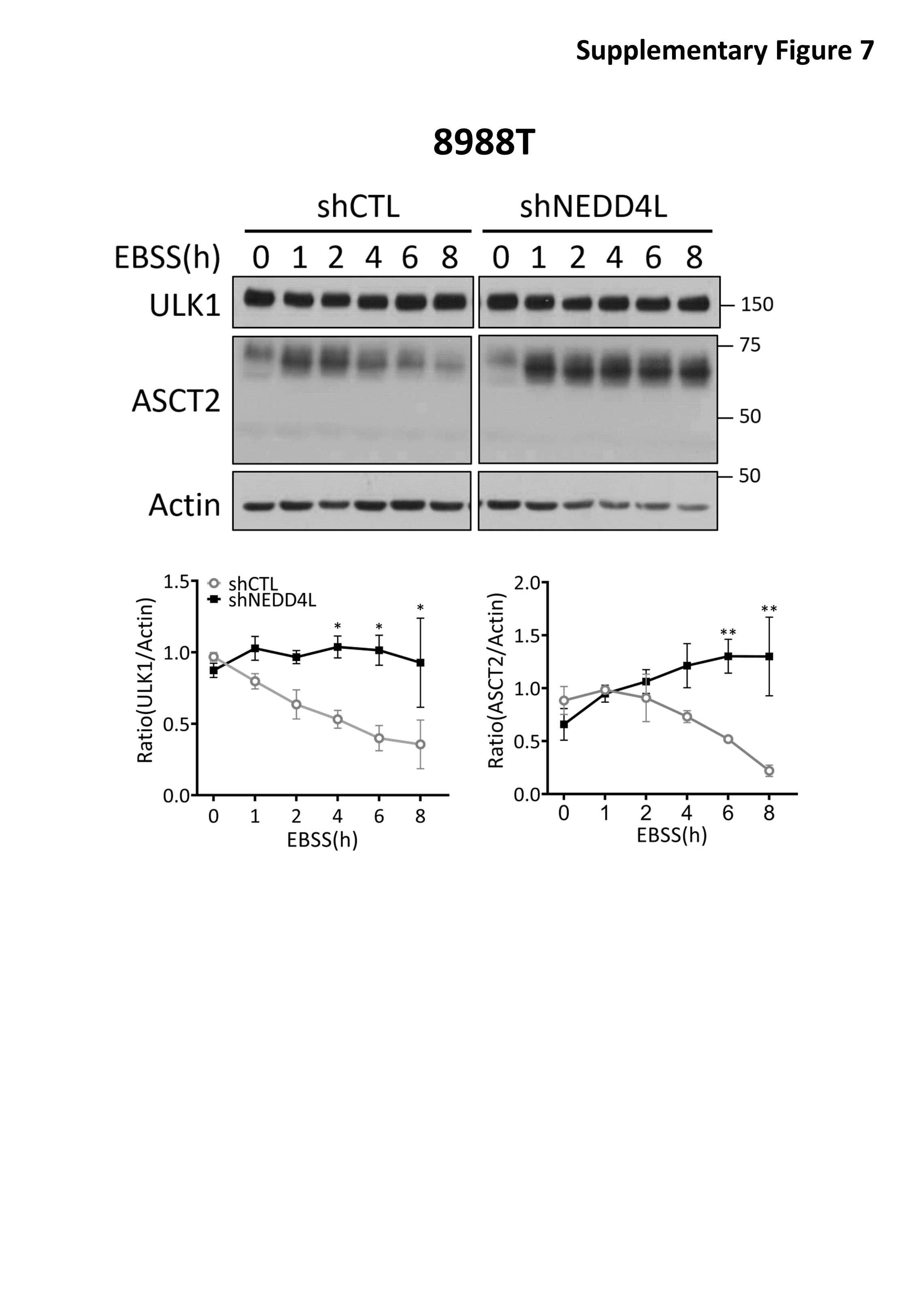

Supplement: Supplementary file 9 — Supplementary Figure 7 [file 41419_2020_2242_MOESM9_ESM.tif]

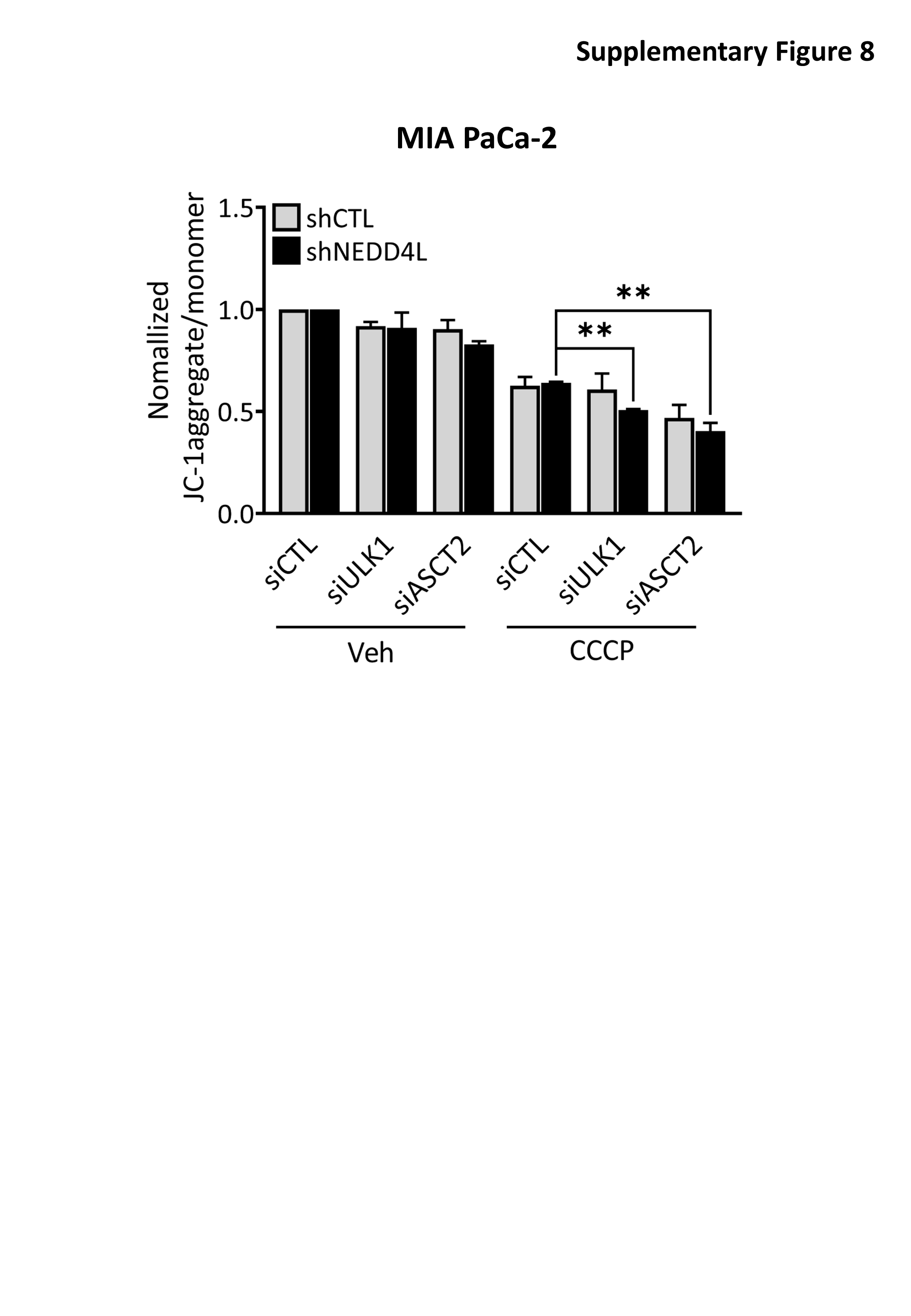

Supplement: Supplementary file 10 — Supplementary Figure 8 [file 41419_2020_2242_MOESM10_ESM.tif]

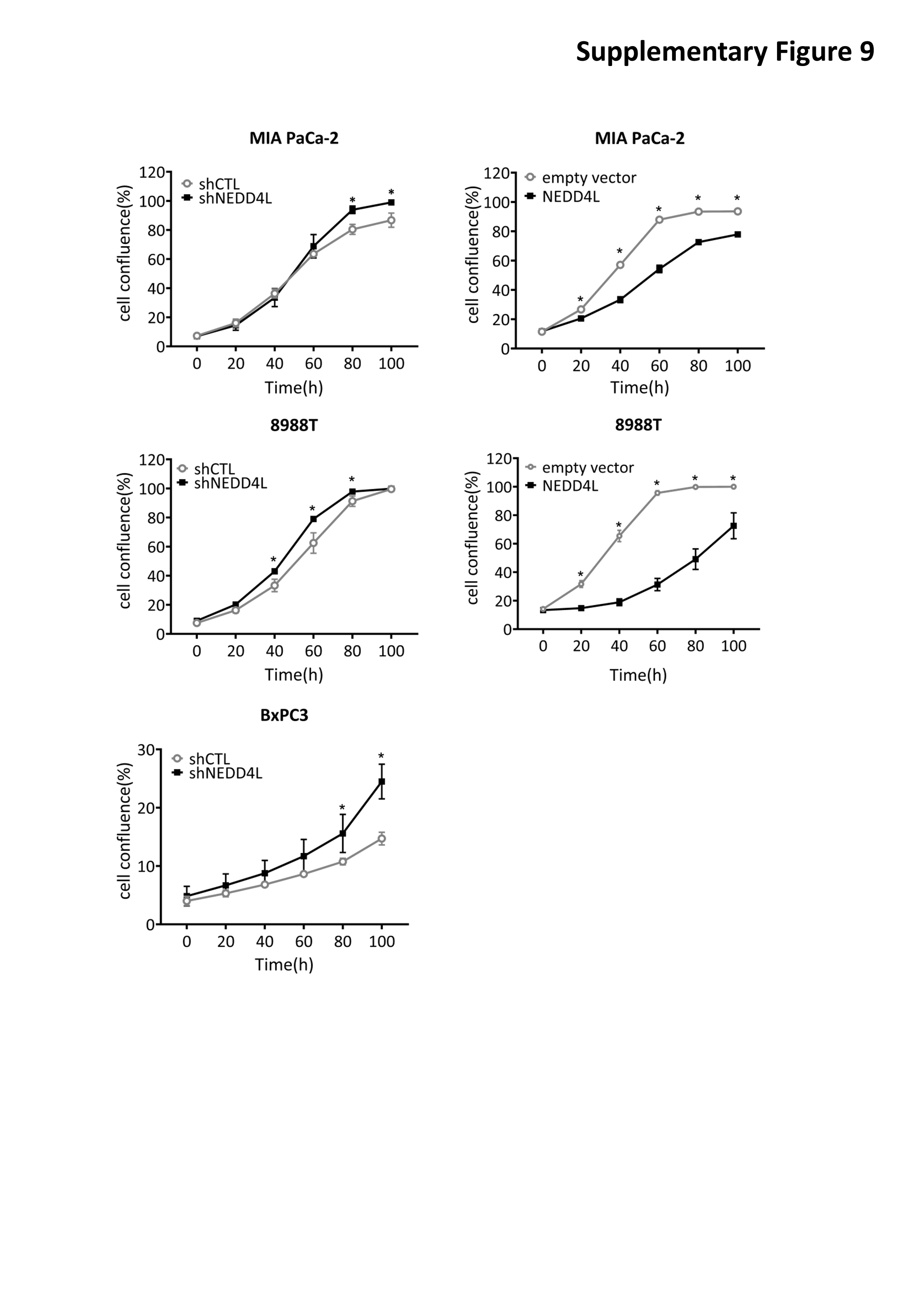

Supplement: Supplementary file 11 — Supplementary Figure 9 [file 41419_2020_2242_MOESM11_ESM.tif]

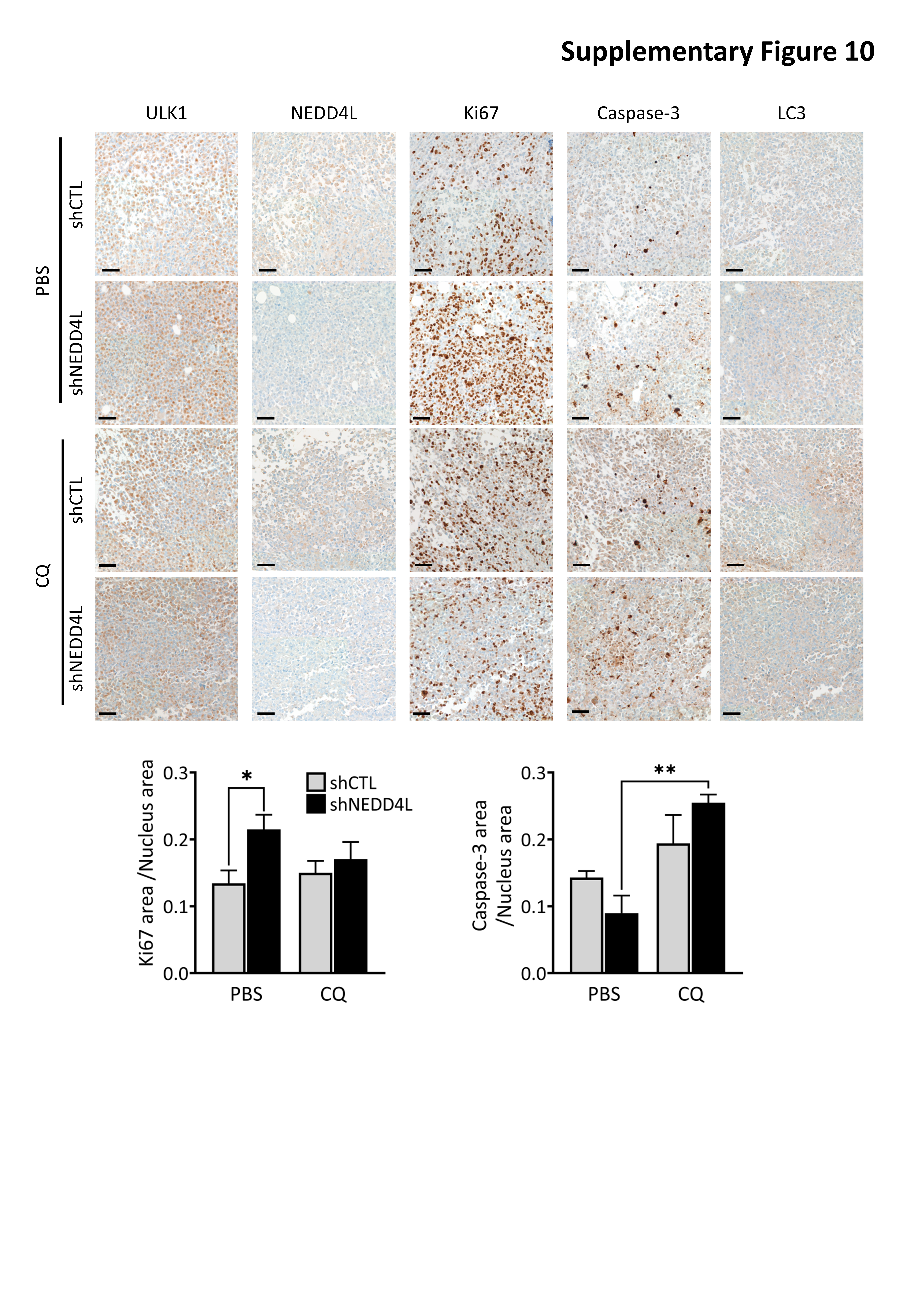

Supplement: Supplementary file 12 — Supplementary Figure 10 [file 41419_2020_2242_MOESM12_ESM.tif]
